# Pruritus and health-related quality of life in chronic liver disease: a longitudinal, survey-based cohort study

**DOI:** 10.1136/bmjgast-2025-001809

**Published:** 2025-12-11

**Authors:** Usha Gungabissoon, Jake Hunnicutt, Eleanor J McDermott, Andrew Lovley, Monica S Frazer, Kaitlin M LaGasse, Mark Kosinski, Kristen L McCausland, Ashleigh McGirr, Helen T Smith, Gideon M Hirschfield, Palak Trivedi

**Affiliations:** 1 Global Epidemiology (Global Regulatory, Safety & Quality), GSK, London, UK; 2 Epidemiology (Global Regulatory, Safety & Quality), GSK, Collegeville, Pennsylvania, USA; 3 VEO Study Delivery, Global Clinical Operations, GSK, Stevenage, UK; 4 Consulting Science, QualityMetric, an IQVIA Business, Providence, Rhode Island, USA; 5 Epidemiology (Global Regulatory, Safety & Quality), GSK, Mississauga, Ontario, Canada; 6 Value Evidence & Outcomes, GSK, London, UK; 7 The Autoimmune and Rare Liver Disease Programme, Division of Gastroenterology and Hepatology, Toronto General Hospital, Toronto, Ontario, Canada; 8 Liver Unit, University Hospitals Birmingham NHS Foundation Trust, Birmingham, UK; 9 National Institute for Health and Social Care Research (NIHR) Birmingham Biomedical Research Centre (BRC), Centre for Liver and Gastrointestinal Research, University of Birmingham, Birmingham, UK; 10 Institute of Immunology and Immunotherapy, University of Birmingham, UK; 11 Institute of Applied Health Research, University of Birmingham, UK

**Keywords:** primary biliary cirrhosis, chronic liver disease, nonalcoholic steatohepatitis, quality of life, primary sclerosing cholangitis

## Abstract

**Objective:**

Cholestatic pruritus is commonly reported in primary biliary cholangitis (PBC) and primary sclerosing cholangitis (PSC); however, information on pruritus in other chronic liver diseases (CLDs) is limited. This survey-based cohort study characterised the severity, persistence and impact of pruritus in PBC, PSC, chronic hepatitis B or C virus infection (HBV/HCV), drug-induced liver injury, autoimmune hepatitis (AIH) and metabolic dysfunction-associated steatohepatitis (MASH). Here, we focus on groups that recruited the most participants: PSC, MASH and HCV. Results are presented in the context of PBC.

**Methods:**

Adults with a CLD of interest from the USA, the UK, Canada and Germany were screened for the presence of pruritus via the worst-itch numerical rating scale (WI-NRS, 3-month recall) between January 2021 and January 2022. Enrolled participants (single liver disease, non-transplant recipients) self-reporting pruritus with no extrahepatic causes in the past 3 months (WI-NRS≥1) were eligible for further health-related quality-of-life (HRQoL) assessments, including WI-NRS (2-week recall), 5-dimensional itch scale and version two of the 36-Item Short-Form Health Survey. Questionnaires were administered at baseline (month 0), month 3 and month 6.

**Results:**

Of 717 screened participants, 40.4% (AIH)–72.7% (HCV) reported any pruritus. Of 403 eligible participants, 357 were enrolled. Time since onset of pruritus to enrolment ranged from 28.0 (MASH) to 77.5 months (PBC). Baseline WI-NRS scores ranged from 3.8 (MASH) to 5.1 (PSC). The most selected terms used to describe pruritus across all CLDs were ‘deep itch’ and ‘urgent itch’ (range: 53.7% (HCV)–77.0% (PSC)). Participants with more severe pruritus had worse HRQoL. Intimate relationships, emotional well-being and ability to concentrate were negatively impacted by pruritus. Pruritus persisted over the 6-month study period across all CLDs.

**Conclusion:**

Our study highlights the burden of pruritus experienced by participants across CLDs, highlighting a need to improve symptom recognition and treatments focused on improving HRQoL.

**WHAT IS ALREADY KNOWN ON THE TOPIC**

- Pruritus is a common condition experienced by patients with cholestatic liver diseases such as primary biliary cholangitis (PBC) and primary sclerosing cholangitis (PSC) and is linked to poor quality of life (QoL) and emotional well-being.
- Although pruritus can occur in other chronic liver diseases (CLDs), less is known about its prevalence, persistence, severity, experience and impact on patients’ lives.

**WHAT THIS STUDY ADDS**

- Our study highlights the burden and persistence of pruritus experienced by patients with CLD over time.
- Participants with more severe pruritus had worse health-related QoL; intimate relationships, emotional well-being and the ability to concentrate were all negatively impacted by pruritus.
- Where present, pruritus persisted across all CLDs: longitudinal analyses revealed that most participants with moderate-to-severe pruritus at baseline still reported moderate-to-severe pruritus over the 6-month period of study evaluation.

**HOW THIS STUDY MIGHT AFFECT RESEARCH, PRACTICE OR POLICY**

- Our study highlights that increased clinician awareness, engagement and research is required to address the burden of pruritus experienced by patients across CLDs, and understand the drivers of pruritus occurrence in patients, the therapeutic shortfall in an era of current anti-itch treatments and the factors influencing persistence versus resolution of symptoms over time.

## Introduction

 Pruritus is commonly experienced by patients with cholestatic liver diseases, such as primary biliary cholangitis (PBC) and primary sclerosing cholangitis (PSC).^1^,^5^ Pruritus of any severity is estimated to affect 55%–89% of patients with PBC, with approximately 25%–53% of patients with PBC reporting moderate-to-severe pruritus or clinically significant pruritus.^1 3 4 6^ Approximately 20%^2 5 7^ of those with PSC experience moderate-to-severe pruritus.^4 8^ In PBC specifically, persistent pruritus (experienced frequently or all the time since the development of PBC), despite current medical therapies, has been reported in over one-third of patients.^3^ Pruritus is linked to other elements of poor health-related quality of life (HRQoL) and emotional well-being, including sleep disturbance, fatigue and cognitive impairment^34 9^,^13^; in extreme cases, severe pruritus can lead to suicidal ideation or can be an indication for liver transplantation.^9 10^ Despite its recognised impact, pruritus is undercaptured in routine clinical care. Analyses of the PicnicHealth PBC cohort found that almost 50% of patients who self-reported clinically significant pruritus did not have pruritus noted in their medical records.^14^ Moreover, the UK-PBC audit of approximately 9000 patients showed that one-third of patients had not been assessed for symptoms.^15^

Although pruritus can occur in other chronic liver diseases (CLDs),^1016^,^18^ less is known about its prevalence, persistence, severity and impact on patients. This might be compounded by a lack of awareness among patients and physicians,^1016^,^19^ despite pruritus being recognised as a cornerstone of QoL questionnaires in CLD.^20^ Data from Japan indicate that up to 53% of patients with CLDs experience pruritus (irrespective of season and aetiology) according to the Kawashima score and visual analogue scale.^21^ Post hoc analysis of the STELLAR 3 and STELLAR 4 clinical trials in patients with advanced fibrosis due to metabolic dysfunction-associated steatohepatitis (MASH, formerly non-alcoholic steatohepatitis (NASH)) also shows that over 25% of patients experience pruritus according to the Chronic Liver Disease Questionnaire-NASH instrument.^18^ Furthermore, the pathophysiology of pruritus in non-cholestatic diseases is largely unknown; however, mediators such as interleukin-31 have been investigated in MASH.^22^ However, there is limited knowledge on whether pruritus in non-biliary CLDs differs from that observed in PBC and PSC.

The overarching goal of this study was to describe the severity, experience and persistence of pruritus, the differences in management strategies offered by clinicians and the impact of itch on HRQoL across a range of CLDs. Although a wide range of CLDs were included in this study, to permit meaningful evaluations, we focus on the four enrolled CLD cohorts that recruited the greatest number of participants (n≥50): PBC, PSC, MASH and chronic hepatitis C virus infection (HCV).

## Methods

### Study objectives

The primary objective of the study was to describe the severity and persistence of pruritus and its impact on HRQoL prospectively over a 6-month period. In addition, this study sought to retrospectively describe the patient journey of pruritus from its onset to present day (completed at baseline), diurnal and seasonal patterns and descriptors of itch sensations.

### Study design and setting

**Figure 1:**
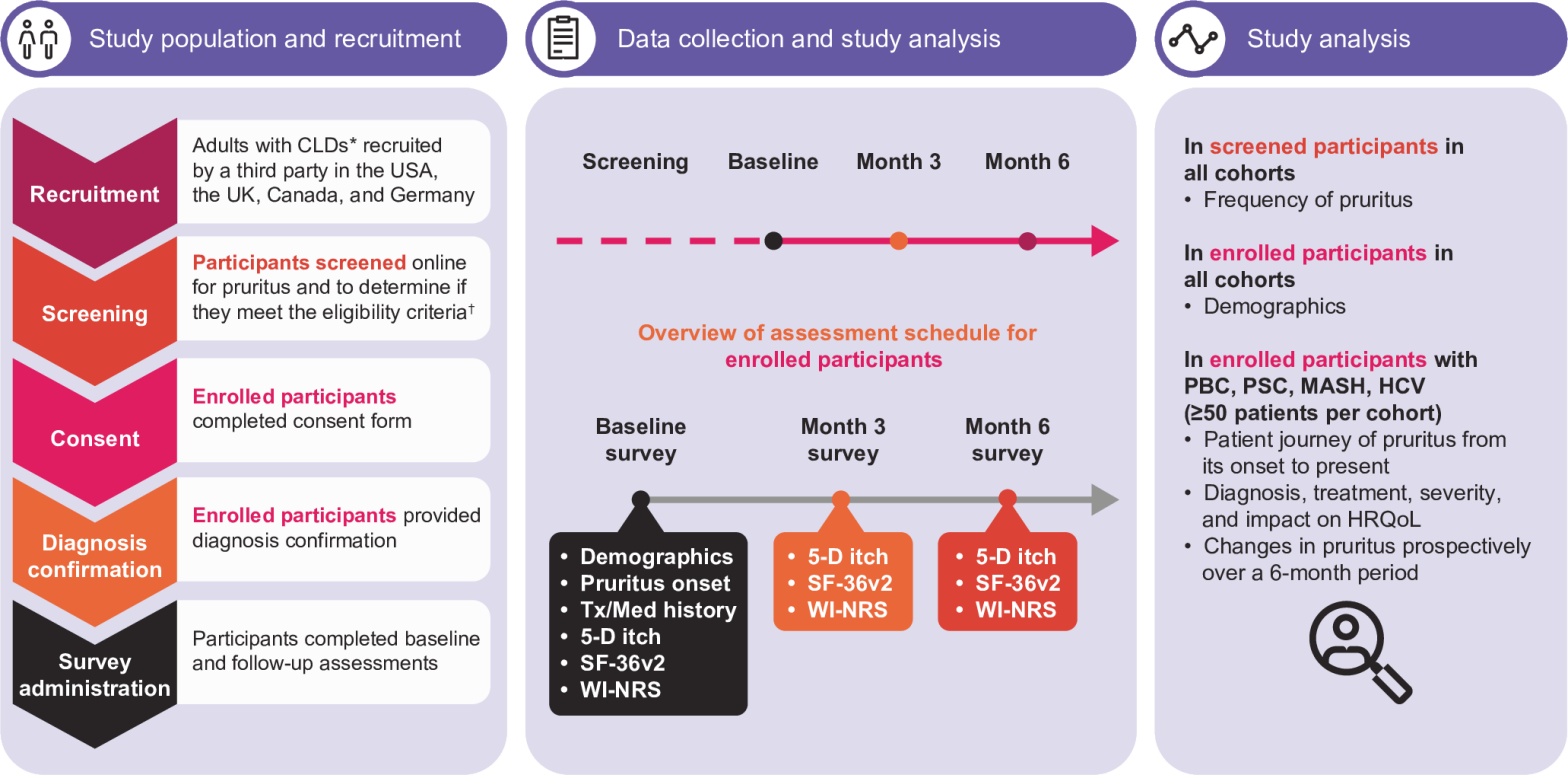
Study design overview. *PBC, PSC, AIH, HBV, HCV, DILI or MASH. †Participants who reported experiencing pruritus in the previous 3 months at screening were invited to participate in the prospective study of pruritus over time if they met the enrolment criteria. 5-D, five-dimensional; AIH, autoimmune hepatitis; CLD, chronic liver disease; DILI, drug-induced liver injury; HBV, chronic hepatitis B virus infection; HCV, chronic hepatitis C virus infection; HRQoL, health-related quality of life; MASH, metabolic dysfunction-associated steatohepatitis; PBC, primary biliary cholangitis; PSC, primary sclerosing cholangitis; SF-36v2, version two of the 36-Item Short-Form Health Survey; Tx/Med, treatment/medical; WI-NRS, worst-itch numerical rating scale.

The study design is described in figure 1. This online, survey-based cohort study was conducted among adults with CLDs and pruritus from Canada, Germany, the UK and the USA. Participants were recruited between January 2021 and January 2022 and followed up over a 6-month period.

### Participants

#### Recruitment and screening

Potential participants were recruited through partnerships with patient groups via newsletters and social media platforms, databases and referrals from healthcare providers (HCPs). Although there were no specific research sites for this study, one site supported participant recruitment in Canada (University Health Network, Professor Gideon Hirschfield), where the programme was advertised to patients. Screened participants and referring clinicians had no knowledge of the pruritus-related objectives that formed the subsequent phases of study; as such, the institutional review board-approved recruitment messages used did not reference the pruritus-related objectives of the study (online supplemental methods). This approach enabled the use of screening data to describe the proportion of screened participants with CLDs that reported pruritus in the previous 3-month period, an exploratory objective of this study. Participants who reported experiencing pruritus during this period at screening were invited to participate in the prospective study of pruritus over time.

Enrolled participants were required to meet the following inclusion criteria: provide consent via an electronic form; able to read and respond to questions in the study language (English or German); ≥18 years of age; live in one of the target countries during the study period; self-report pruritus within the past 3 months (worst-itch numerical rating scale (WI-NRS) ≥1) that was not associated with an insect bite, poison ivy or allergies; provide confirmation of having been diagnosed with, and currently having, one of the following primary CLDs of interest: PBC, PSC, autoimmune hepatitis (AIH), chronic hepatitis B virus infection (HBV), HCV, drug-induced liver injury (DILI) or MASH. Up to five participants with HCV who were either cured or in sustained virological response but were still experiencing pruritus were eligible for enrolment. Importantly, confirmation of CLD aetiology was required to be provided by participants at this stage, either through a note from an HCP, medical records, electronic health records or a photograph of a prescription medication for the treatment of their CLD. Using guidelines developed by the study team, documentation was reviewed by the recruitment service to confirm eligibility prior to enrolment. Further confirmatory evidence was not required (eg, clinical imaging or biopsy findings). For the purposes of this study, in the absence of confirmatory evidence, MASH was considered to be suspected. See online supplemental methods for exclusion criteria.

### Study assessments and outcomes

Surveys were administered at three time points: baseline (month 0), month 3 and month 6. Participants completed the surveys through a secure, web-based electronic data capture system, Confirmit.^23^ At baseline, and on completion of the screening HRQoL questionnaire, enrolled participants were invited to complete further questions on pruritus intensity and to retrospectively recall details of symptoms since onset, including time since onset and their perception of whether pruritus severity had changed since onset. Pruritus-specific questionnaires were used, namely the WI-NRS and the five-dimensional (5-D) itch scale, alongside version two of the 36-Item Short-Form Health Survey (SF-36v2), a QoL assessment tool (see online supplemental methods for full detail of the 5D-itch and SF-36v2 tools). Thereafter, enrolled participants were asked to complete the WI-NRS, the 5-D itch scale and SF-36v2 at month 3 and month 6. Participants received an honorarium for each survey completed.

### Worst-itch numerical rating scale

The WI-NRS is a single-item instrument that asks participants to rate their itch severity at its worst.^24^ It was administered at each time point (baseline, month 3 and month 6), whereby participants were asked to rate their worst itch from the past 2 weeks. A version of the WI-NRS with a 3-month recall was administered to screened participants. The item uses an 11-point scale, with 0 representing ‘no itching’ and 10 representing ‘worst imaginable itching’. For this study, the following WI-NRS cut-offs were used for interpreting itch severity: 0, no itch; 1–3, mild itch; 4–6, moderate itch and 7–10, severe itch.

### Additional survey items

To characterise the patient journey, surveys included a combination of validated patient-reported outcome (PRO) instruments and unique items developed specifically for this study were used. These included items adapted from the itch domain of the Primary Biliary Cholangitis-40 items (PBC-40) and generated from concepts identified in existing literature to measure concepts of interest not included in the validated instruments. Survey items captured participants’ retrospective experience with pruritus in terms of mitigation and exacerbation of itch, medications used to treat itch (full list in online supplemental methods), description of pruritus sensation, itch severity, daily variation in itch, HCP involvement in managing pruritus, changes to lifestyle due to itch and impacts on intimate relationships, emotional well-being and ability to concentrate (adapted items from the PBC-40 itch domain). Descriptors of pruritus were given as a prespecified list of 11 descriptors of itch with an ‘other’ option. Survey items assessing demographics, time of pruritus onset and description of the course of pruritus were administered at baseline only.

### Study size

This non-interventional study was designed to assess objectives through descriptive analyses; therefore, no formal sample size/power calculations were proposed. The results primarily focus on the four CLD groups that recruited the largest number of participants (PBC, PSC, MASH and HCV). Target recruitment sample sizes were initially chosen for the estimation of the frequency of pruritus overall and within each liver disease group; estimations of moderate-to-severe pruritus were added post hoc.

### Statistical methods

All analyses were performed using SAS 9.4. The proportion of individuals reporting pruritus was described in the screened CLD populations. For the main study, analyses were conducted on the enrolled population, ie, those who met the eligibility criteria for the study. Descriptive analyses of the enrolled participants overall and by CLD group were conducted at baseline, month 3 and month 6 time points with stratifications by pruritus severity (WI-NRS scores). Continuous variables were summarised using means (SD) and medians (IQRs); categorical variables were summarised using frequencies and proportions. Aetiology-specific subanalyses were performed for disease groups with >50 enrolled participants. For cross-sectional analyses, tests of omnibus association were conducted across CLD groups; the Kruskal-Wallis test was used for continuous variables. A post hoc analysis using χ^2^ testing focused on patients with moderate-to-severe pruritus across disease groups with sufficient sample size was also included. Where indicated, PBC was used as a comparison group for pairwise tests, where a significant difference across CLD groups was found. All analyses were based on observed data only; missing data were not imputed.

Longitudinal analyses examined patterns of change in WI-NRS, 5D-itch and SF-36v2 scores across CLD groups over time (baseline to month 3 and baseline to month 6) using a series of linear mixed-effects models. Each PRO score of interest was the dependent variable (ie, outcome). In each mixed-effects model, the participant was included as a random intercept and variables for CLD group, study time point and an interaction term for CLD groups by study time were included as fixed effects. Additionally, to adjust for potential confounders selected a priori, age, gender and time since diagnosis were included in each model as time-invariant fixed effects. Unless otherwise specified, the longitudinal analyses reported here used all available data for the relevant analytic sample (ie, if an individual missed one or more surveys, they were still retained in the analyses).

## Results

### Pruritus reported in the screened population

Over a 12-month recruitment period, 717 participants completed the screening survey (table 1). In the overall screened sample, 403 (56%) participants reported pruritus in the past 3 months (WI-NRS ≥1) and 302 (42%) reported moderate-to-severe pruritus in the past 3 months (WI-NRS ≥4). Any degree of pruritus (based on WI-NRS ≥1 with a 3-month recall) was reported in 72.7% of screened participants with HCV, 70.0% with HBV, 69.1% with MASH, 40.4% with AIH and 52.8% with DILI. By comparison, 50.9% and 48.9% of participants with PSC and PBC, respectively, reported pruritus.

**Table 1:** Presence of pruritus (WI-NRS with 3-month recall) among the screened population (all CLDs)

Cohort	N	Reported pruritus in past 3 months (WI-NRS ≥1), n (%)*	Reported moderate-to-severe pruritus in past 3 months (WI-NRS ≥4), n (%)*
PBC	174	85 (48.9)	68 (39.1)
PSC	173	88 (50.9)	65 (37.6)
AIH	99	40 (40.4)	30 (30.3)
MASH	97	67 (69.1)	45 (46.4)
HCV	77	56 (72.7)	40 (51.9)
HBV	50	35 (70.0)	26 (52.0)
DILI†	36	19 (52.8)	13 (36.1)

Tables are ordered by the size of the screened CLD populations studied. The proportions represent the percentages of the screened population who reported experiencing pruritus within the past 3 months. As these data are derived from a convenience sample and do not provide formal prevalence estimates, CIs are not presented. The available sample size may not be sufficient for a reliable estimate of the frequency of moderate-to-severe pruritus, and interpretations should be made with caution.

* Derived based on WI-NRS (on a 0–10 scale) with a 3-month recall period administered at screening, before eligibility criteria were applied.

† This included both cholestatic and mixed drug-induced liver injury patient populations.

AIH, autoimmune hepatitis; CLD, chronic liver disease; DILI, drug-induced liver injury; HBV, chronic hepatitis B virus infection; HCV, chronic hepatitis C virus infection; MASH, metabolic dysfunction-associated steatohepatitis; PBC, primary biliary cholangitis; PSC, primary sclerosing cholangitis; WI-NRS, worst-itch numerical rating scale.

### Demographics of enrolled participants

Of the 717 participants screened, 403 were eligible, of whom 357 completed the baseline survey, met all eligibility criteria including confirmation of CLD diagnosis and self-reported pruritus, and were enrolled (online supplemental figure S1). Of 403 individuals deemed eligible to participate, 46 participants did not complete the baseline survey. Participants with PBC, PSC, MASH and HCV comprised the majority of enrolled participants (>50 in each group), and thus data from these cohorts were subjected to further analyses presented henceforth. Notably, women comprised the predominant demographic in the PBC and PSC groups, whereas there were similar proportions of men and women in the HCV and MASH groups (table 2). The mean (SD) age of the overall sample was 51.3 (12.8) years (mean age range: 44.1–57.1 years across CLDs), with the majority of participants recruited from the USA (41.7%) and Germany (30.3%; online supplemental table S1). Participants with MASH were more recently diagnosed with their respective disease (4.8 (3.9) years) than participants with PBC, PSC or HCV.

**Table 2:** Baseline demographics and pruritus characteristics of patients with PBC, PSC, MASH or HCV

Characteristic	PBC (n=80)	PSC (n=74)	MASH (n=61)	HCV* (n=54)
Age, years				
Mean (SD)	57.1 (10.6)	47.3 (14.9)	54.0 (9.9)	49.5 (11.7)
Median (IQR)	57.0 (15.5)	47.5 (25.0)	55.0 (11.0)	48.5 (17.0)
Time since chronic liver disease diagnosis, years†				
Mean (SD)	6.3 (5.9)	6.3 (5.7)	4.8 (3.9)	8.4 (8.4)
Median (IQR)	4.5 (7.0)	5.0 (7.0)	4.0 (4.0)	5.0 (8.0)
Sex, n (%)				
Female	71 (88.8)	46 (62.2)	34 (55.7)	25 (46.3)
Male	9 (11.3)	28 (37.8)	27 (44.3)	29 (53.7)
Country, n (%)				
UK	25 (31.3)	29 (39.2)	1 (1.6)	0
USA	24 (30.0)	22 (29.7)	44 (72.1)	37 (68.5)
Canada	16 (20.0)	8 (10.8)	0	1 (1.9)
Germany	15 (18.8)	15 (20.3)	16 (26.2)	16 (29.6)
Time since itch onset, months				
Mean (SD)	77.5 (106.0)	62.2 (63.0)	28.0 (31.6)	35.8 (33.0)
Median (IQR)	45.5 (68.5)	32.0 (83.0)	15.0 (30.0)	28.5 (33.0)
Compared with the time when your itch/pruritus began, how would you describe the severity of your itch/pruritus now? (n (%))				
Much less severe now	9 (11.3)	11 (14.9)	8 (13.1)	5 (9.3)
Somewhat less severe now	16 (20.0)	10 (13.5)	6 (9.8)	4 (7.4)
About the same	35 (43.8)	24 (32.4)	28 (45.9)	25 (46.3)
Somewhat more severe now	11 (13.8)	23 (31.1)	16 (26.2)	15 (27.8)
Much more severe now	9 (11.3)	6 (8.1)	3 (4.9)	5 (9.3)
Compared with the time when your itch/pruritus began, would you say your itch/pruritus happens more or less frequently now? (n (%))				
Much less frequently now	9 (11.3)	6 (8.1)	7 (11.5)	6 (11.1)
Somewhat less frequently now	14 (17.5)	16 (21.6)	10 (16.4)	3 (5.6)
About the same	29 (36.3)	19 (25.7)	27 (44.3)	28 (51.9)
Somewhat more frequently now	19 (23.8)	21 (28.4)	13 (21.3)	11 (20.4)
Much more frequently now	9 (11.3)	12 (16.2)	4 (6.6)	6 (11.1)
Over the past 2 weeks, on average, how often did you experience itch/pruritus? (n (%))				
Not at all	5 (6.3)	7 (9.5)	1 (1.6)	0 (0.0)
1 day per week	13 (16.3)	15 (20.3)	11 (18.0)	5 (9.3)
2–3 days per week	11 (13.8)	15 (20.3)	20 (32.8)	20 (37.0)
4–6 days per week	25 (31.3)	13 (17.6)	11 (18.0)	8 (14.8)
Every day	26 (32.5)	24 (32.4)	18 (29.5)	21 (38.9)
Over the past 2 weeks, how often have you scratched so much that it made your skin raw? (n (%))				
Not at all	42 (52.5)	36 (48.6)	36 (59.0)	28 (51.9)
1 day per week	11 (13.8)	16 (21.6)	15 (24.6)	11 (20.4)
2–3 days per week	18 (22.5)	11 (14.9)	8 (13.1)	8 (14.8)
4–6 days per week	6 (7.5)	5 (6.8)	1 (1.6)	3 (5.6)
Every day	3 (3.8)	6 (8.1)	1 (1.6)	4 (7.4)
5-D itch scale score,^37^ mean (SD)‡				
Total (5–25)	13.54 (3.93)	13.58 (4.20)	12.02 (3.21)	12.67 (3.80)
Direction (1–5)	3.64 (0.86)	3.57 (1.09)	3.57 (0.78)	3.72 (0.76)
Disability§	3.06 (1.30)	3.00 (1.30)	2.51 (1.23)	2.69 (1.40)
Degree (1–5)	2.73 (0.87)	2.69 (0.87)	2.62 (0.73)	2.72 (0.76)
Distribution¶	2.33 (1.18)	2.59 (1.20)	1.92 (0.92)	1.80 (0.83)
Duration (1–5)	1.79 (1.13)	1.73 (1.10)	1.39 (0.88)	1.74 (1.26)
5-D itch scale score,^37^ median (IQR)‡				
Total (5–25)	13.00 (5.00)	14.00 (6.00)	11.00 (4.00)	11.50 (6.00)
Direction (1–5)	4.00 (1.00)	4.00 (1.00)	4.00 (1.00)	4.00 (1.00)
Disability§	3.00 (2.00)	3.00 (2.00)	2.00 (2.00)	2.00 (2.00)
Degree (1–5)	3.00 (1.00)	3.00 (1.00)	2.00 (1.00)	3.00 (1.00)
Distribution¶	2.00 (2.00)	2.00 (1.00)	2.00 (1.00)	2.00 (1.00)
Duration (1–5)	1.00 (1.00)	1.00 (1.00)	1.00 (0.00)	1.00 (1.00)
WI-NRS score, 2-week recall				
Mean (SD)	4.99 (2.81)	5.12 (2.74)	3.77 (2.28)	4.56 (2.44)
Median (IQR)	5.00 (5.00)	5.00 (5.00)	3.00 (3.00)	4.00 (5.00)
WI-NRS category, 2-week recall (n (%))				
No itch	4 (4.88)	2 (2.63)	0 (0)	0 (0.0)
Mild (1–3)	25 (30.49)	24 (31.58)	31 (50.82)	22 (40.74)
Moderate (4–6)	25 (30.49)	22 (28.95)	21 (34.43)	18 (33.33)
Severe (7–10)	26 (31.71)	26 (34.21)	9 (14.75)	14 (25.93)

Due to rounding, percentages may not add to 100%.

* Four participants with HCV who were either cured or in sustained virological response, but were still experiencing pruritus, were enrolled.

† At screening, the approximate year of CLD diagnosis was captured. Time since diagnosis was calculated as the difference (in years) between screening year and year of diagnosis.

‡ Higher 5-D itch scale scores indicate higher pruritus severity.

§ The disability domain includes four items that assess the impact of pruritus on daily activities: sleep, leisure/social activities, housework/errands and work/school; the score is achieved by taking the highest score on any of the four items.^37^

¶ For the distribution domain score, the number of affected body parts is tallied (potential sum 0–16) and split into five to delineate the score: sum of 0–2=score of 1, sum of 3–5=score of 2, sum of 6–10=score of 3, sum of 11–13=score of 4 and sum of 14–16=score of 5.^37^

5-D, five dimension; CLD, chronic liver disease; HCV, chronic hepatitis C virus infection; MASH, metabolic dysfunction-associated steatohepatitis; PBC, primary biliary cholangitis; PSC, primary sclerosing cholangitis; WI-NRS, worst-itch numerical rating scale.

### Retrospective recall of pruritus experience and onset

The mean time since pruritus onset to enrolment ranged from 28.0 months (MASH) to 77.5 months (PBC; table 2). Overall, >32% of participants across CLDs reported their pruritus severity was ‘about the same’ since it began (table 2), with >25% indicating that their pruritus was ‘somewhat more severe now’ or ‘much more severe now’ than at onset. More specifically, 44.6% of participants with PSC indicated that their pruritus occurred ‘somewhat more frequently now’ or ‘much more frequently now’ than its frequency at onset, compared with other CLDs (range for other CLD groups: 27.9% (MASH) to 35.0% (PBC)). When asked to recall frequency of pruritus over the previous 2 weeks, 29.5% (MASH) to 38.9% (HCV) of participants across CLDs reported experiencing pruritus every day (table 2), with a similar proportion of participants reporting pruritus on 1–3 days per week (range: 30.0% (PBC) to 50.8% (MASH)). Between 16.4% (MASH) and 33.8% (PBC) of participants said that on average, they scratched their skin enough to make it raw on at least 2 days each week (table 2).

When describing pruritus, the terms ‘deep itch’ and ‘urgent itch’ were selected (from a prespecified list of descriptors) by the highest percentage of participants across CLDs (range: 53.7% (HCV) to 77.0% (PSC); online supplemental table S2). To characterise diurnal patterns, participants reflected on when pruritus was at its worst. Within CLDs, over half of participants with HCV reported pruritus was worse during the day, whereas over half of participants with PBC or PSC reported itch was worse at night and 41.0% of participants with MASH reported it was ‘about the same during the day and night’ (online supplemental table S2). There were no notable seasonality patterns, with the majority of participants reporting their pruritus was the same across seasons (range: 50.8% (MASH) to 61.3% (PBC)).

### Impact of pruritus and management strategies tried at baseline

In terms of how HCPs approached pruritus, the majority (range: 60.0% (PBC) to 75.7% (PSC)) of participants reported that their HCP spoke with them about pruritus. Participants with HCV and MASH commonly reported that their physician looked at their skin; however, this was less common in PBC/PSC. Participants who reported that their HCP had not talked to them about pruritus ranged from 5.4% in PSC to 23.0% in MASH (online supplemental table S3).

Across CLDs, just under a third of participants reported making no lifestyle adjustments since the onset of pruritus. Of those who altered their lifestyle, the two most common adjustments were changes to clothing (ranging from 33.3% (HCV) to 43.8% (PBC)) and changes in diet (31.1% (MASH and PSC) to 36.3% (PBC); online supplemental table S4). In terms of pruritus management since onset, the highest proportion of participants who had ever tried prescription medications for their pruritus was in the PSC group (70.3%). Across CLDs, in participants who had tried prescription medications for their pruritus, participants most frequently reported that their itch had ‘somewhat improved’ (range: 12.5% (PBC) to 25.7% (PSC)). The most common treatments used were topical remedies (range: 73.8% (PBC and MASH) to 82.4% (PSC)), antihistamines (range: 38.9% (HCV) to 73.0% (PSC)) and bile acid sequestrants (range: 1.9 (HCV) to 48.6% (PSC); online supplemental table S3). Approximately two-thirds of the PSC group (66.2%) had tried over-the-counter antihistamines, with 23% reporting no improvement (online supplemental table S3). Over 70% of participants across CLDs reported that scratching either did not improve their pruritus at all or only slightly improved it (online supplemental table S3).

**Figure 2:**
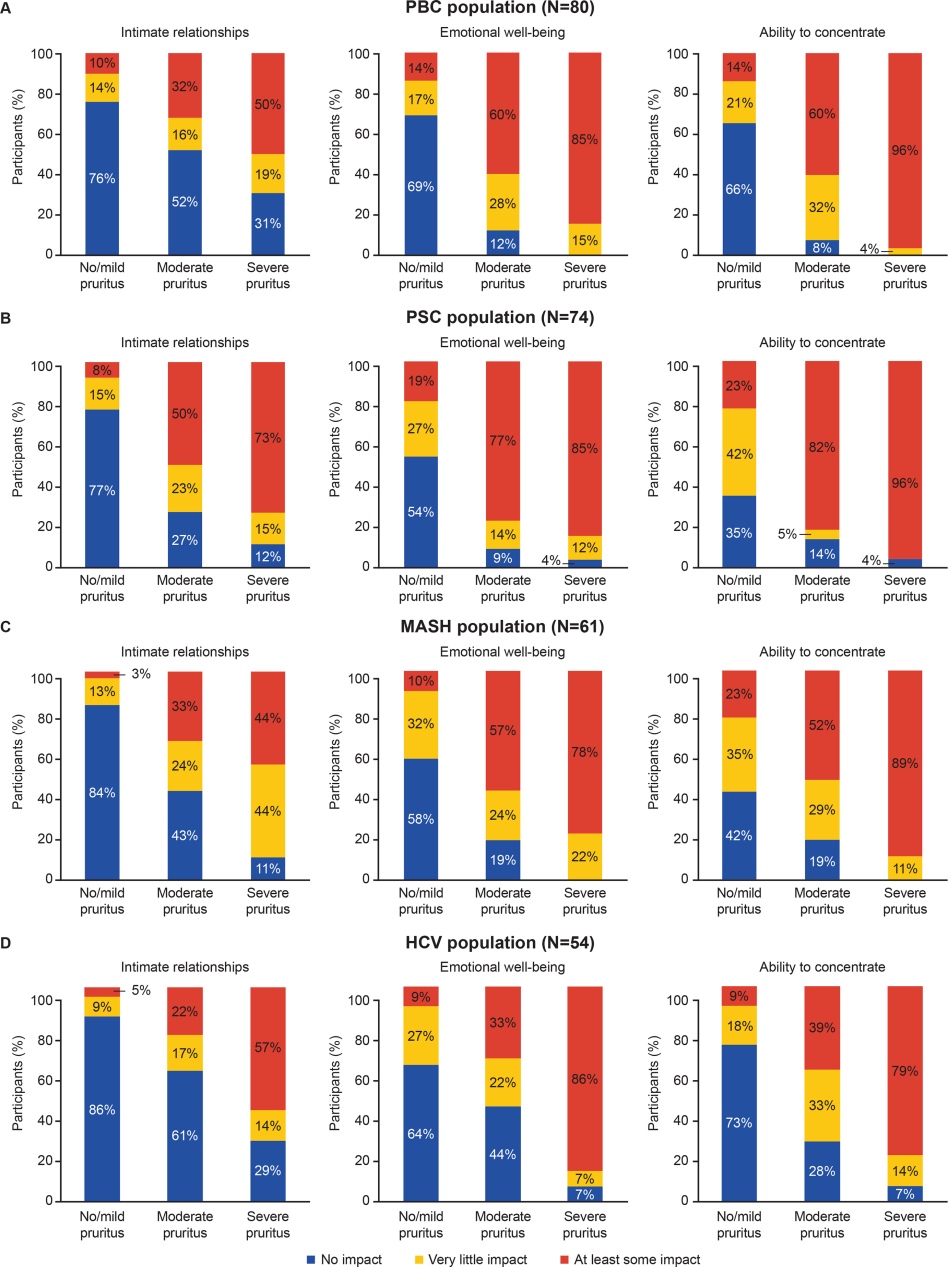
Impact of pruritus on emotional well-being, ability to concentrate and intimate relationships at baseline by pruritus severity in participants with PBC (**A**), PSC (**B**), MASH (**C**) and HCV (**D**). The wording of the survey question was as follows: “Over the past 2 weeks, when your itch/pruritus was at its worst, please indicate how much each aspect of your life was impacted by itch/pruritus”. Impact was scored as the following: no impact, 1; very little impact, 2; some impact, 3; quite a bit of impact, 4; a great deal of impact, 5 (for the figure above, the last three categories were combined to form the ‘at least some impact’ category above (displaying scores from 3 to 5)). Pruritus severity was determined by WI-NRS: no/mild itch, 0–3; moderate itch, 4–6; severe itch, 7–10. HCV, chronic hepatitis C virus infection; MASH, metabolic dysfunction-associated steatohepatitis; PBC, primary biliary cholangitis; PSC, primary sclerosing cholangitis; WI-NRS, worst-itch numerical rating scale.

Intimate relationships, emotional well-being and ability to concentrate over the last 2 weeks were all impacted by pruritus and appeared to worsen with increased severity of itch in every disease group (figure 2).

### Patient-reported outcome scores at baseline

#### Pruritus

Mean (SD) WI-NRS scores at baseline ranged from 3.8 (2.28) for MASH to 5.1 (2.74) for PSC (table 2). Pairwise comparisons also showed that mean pruritus scores in PBC were significantly greater than mean scores in MASH (p=0.009). Similar results were seen in pairwise tests, where WI-NRS scores were treated as categorical variables (mild/moderate/severe pruritus). In line with these results, post hoc examinations showed that a higher proportion of patients with PBC experienced moderate-to-severe itch when compared with patients with MASH. No other pairwise differences between CLD groups were identified. The proportion of participants reporting moderate-to-severe pruritus across CLDs ranged from 49.2% (MASH) to 64.9% (PSC).

Mean domain scores on the 5-D itch scale were similar across CLDs (table 2). Mean duration domain scores ranged from 1.4 (MASH) to 1.8 (PBC), which corresponded to experiencing pruritus for 0–12 hours per day. Mean scores for the degree domain ranged from 2.6 (MASH) to 2.7 (PBC), describing pruritus that is approaching moderate intensity. Distribution domain scores, representing the number of body parts affected by pruritus, ranged from 1.8 (indicating up to two body parts; HCV) to 2.6 (indicating three to five body parts; PSC). Participants across CLDs reported a score of 3–<4 for the direction domain, indicating that pruritus was slightly better but still present or unchanged since onset. The mean total score (SD) for the 5-D itch scale ranged from 12.0 (3.21) in MASH to 13.6 (4.20) in PSC, within a possible range of 5 (no pruritus) to 25 (most severe pruritus).

Pairwise tests indicated that participants with PBC (mean (SD); 2.3 (1.18)) scored significantly higher in the 5-D itch distribution domain (more body areas affected by pruritus) than participants with HCV (1.8 (0.83); p=0.011) or MASH (1.9 (0.92); p=0.044). The pairwise tests showed that participants with PBC also scored significantly higher (worse) than participants with MASH on both 5-D itch disability (3.1 (1.30) vs 2.5 (1.23), respectively; p=0.011) and total domain scores (13.5 (3.93) vs 12.0 (3.21), respectively; p=0.012). Subanalysis by pruritus severity (WI-NRS) to explore any between-group differences in the 5-D itch mean and individual domain scores revealed that 5-D itch domain scores were higher with increased severity of pruritus (online supplemental figure S2).

#### Health-related quality of life

Overall, HRQoL scores, as measured by the SF-36v2, were similar between CLDs (table 3). The largest deficits were observed in the general health domain, ranging from 36.5 (PSC) to 38.3 (PBC; table 3). When mean SF-36v2 scores were grouped by pruritus severity, the lowest scores (indicating worse health status) were among participants in the severe pruritus category and highest (better health status) among participants in the no/mild itch category (online supplemental figure S3).

**Table 3:** Baseline SF-36v2 scores in patients with PBC, PSC, MASH or HCV

SF-36v2 domain score	PBC (n=80)		PSC (n=74)		MASH (n=61)		HCV (n=54)	
	Mean (SD)	Median (IQR)	Mean (SD)	Median (IQR)	Mean (SD)	Median (IQR)	Mean (SD)	Median (IQR)
Physical functioning	45.61 (10.00)	47.95 (15.43)	47.69 (9.74)	51.81 (11.57)	43.81 (11.44)	46.02 (19.29)	42.74 (12.35)	44.10 (19.29)
Role physical	41.35 (10.41)	41.71 (16.51)	44.09 (10.05)	46.11 (15.42)	43.40 (12.09)	43.91 (26.42)	43.58 (11.79)	45.01 (22.02)
Bodily pain	42.79 (8.37)	41.92 (9.08)	46.97 (10.45)	45.87 (13.02)	43.87 (9.62)	45.47 (13.03)	43.21 (9.80)	41.73 (13.03)
General health	38.34 (8.74)	37.17 (11.91)	36.47 (7.76)	36.07 (9.7)	37.50 (9.91)	37.61 (14.12)	37.42 (10.09)	37.61 (15.44)
Vitality	40.61 (8.92)	40.57 (13.61)	42.45 (10.05)	41.93 (16.33)	43.98 (11.44)	44.65 (16.33)	43.14 (10.17)	41.93 (13.61)
Social functioning	43.45 (11.73)	46.85 (19.77)	44.31 (10.80)	46.85 (14.82)	44.83 (12.96)	46.85 (19.77)	40.90 (12.89)	41.91 (24.71)
Role emotional	42.04 (11.49)	44.19 (17.18)	43.16 (12.19)	44.19 (22.90)	44.13 (14.71)	48.00 (15.27)	43.83 (14.65)	51.82 (22.90)
Mental health	45.83 (8.71)	45.33 (12.38)	45.77 (10.31)	47.81 (14.86)	47.28 (11.66)	50.29 (19.82)	43.45 (13.03)	42.85 (22.30)
Physical component summary	42.13 (8.75)	42.00 (14.28)	44.68 (9.76)	46.23 (11.68)	41.59 (10.12)	42.88 (15.60)	41.87 (12.10)	45.25 (19.03)
Mental component summary	43.51 (9.77)	45.85 (16.06)	43.43 (10.82)	44.91 (15.95)	46.35 (12.71)	49.83 (17.13)	43.40 (13.66)	46.25 (19.03)

Higher SF-36v2 scores indicate better health-related quality of life.

HCV, chronic hepatitis C virus infection; MASH, metabolic dysfunction-associated steatohepatitis; PBC, primary biliary cholangitis; PSC, primary sclerosing cholangitis; SF-36v2, version two of the 36-Item Short-Form Health Survey.

### Longitudinal analyses of WI-NRS scores

**Figure 3:**
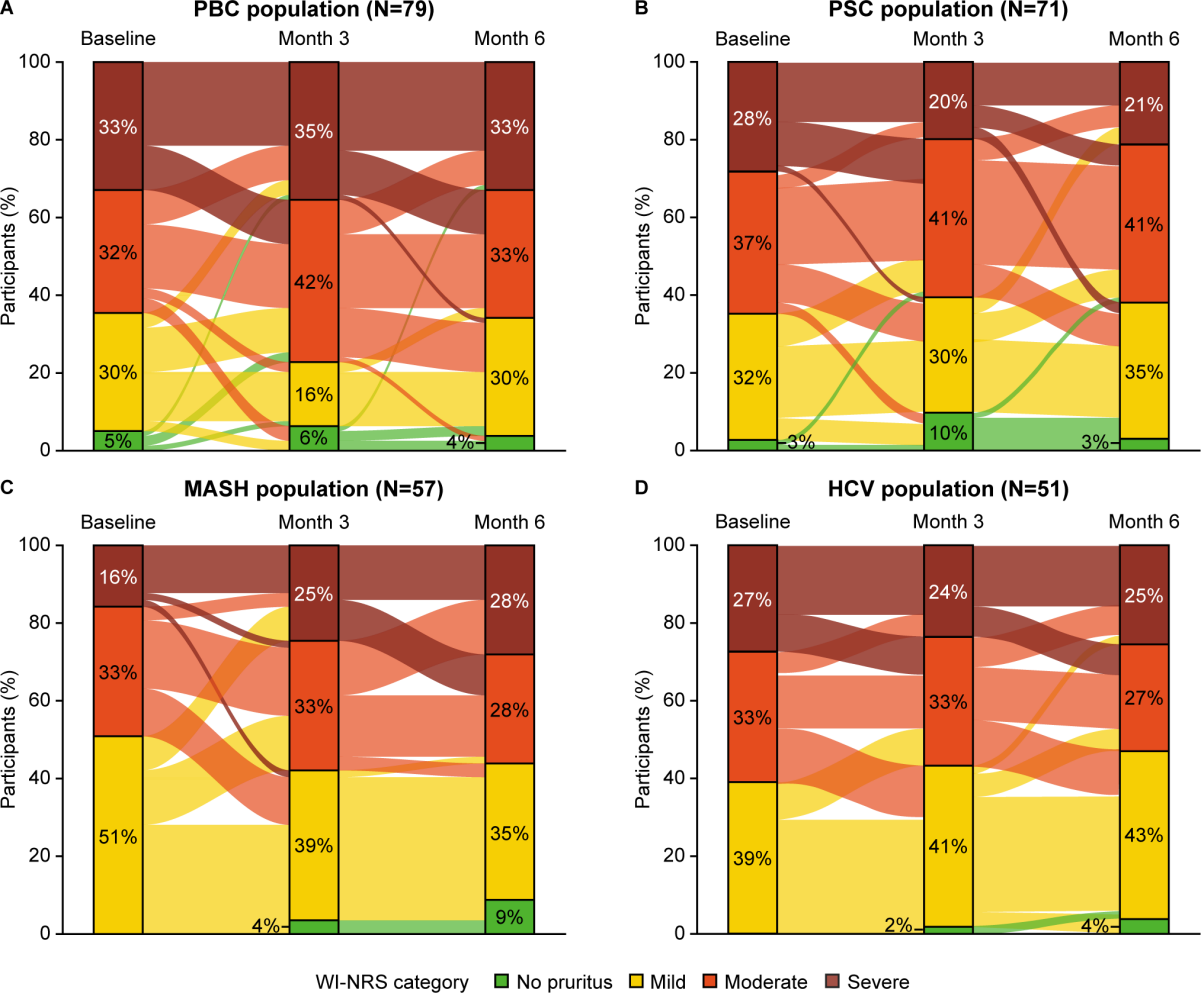
Sankey diagram showing change in pruritus severity over time in (**A**) PBC, (**B**) PSC, (**C**) MASH, (**D**) HCV. The stacked bars indicate the proportion of participants within each WI-NRS category at each time point. The connecting lines represent the direction of change between time points, that is, the proportion of participants moving to a different WI-NRS category or remaining in the same category. Thicker lines indicate more participants. Figures show participants with complete data at all three time points; one participant in the PBC cohort, three participants in the PSC cohort, four participants in the MASH cohort and three participants in the HCV cohort had missing data at ≥1 time point. Numbers may not sum to 100% due to rounding. Pruritus severity was determined by WI-NRS: no itch, 0; mild itch, 1–3; moderate itch, 4–6; severe itch, 7–10. HCV, chronic hepatitis C virus infection; MASH, metabolic dysfunction-associated steatohepatitis; PBC, primary biliary cholangitis; PSC, primary sclerosing cholangitis; WI-NRS, worst-itch numerical rating scale.

The proportion of participants reporting changes in pruritus severity from baseline to month 6 was not significantly different between CLD groups (figure 3; online supplemental figure S4). Longitudinal analyses revealed that across CLDs, most participants with moderate-to-severe pruritus at baseline still had moderate-to-severe pruritus over the 6-month period of study evaluation. This was highest in those with PBC (range across time points: 65%–77%), followed by PSC (61%–65%), HCV (52%–60%) and MASH (49%–58%; figure 3; online supplemental figure S4). For WI-NRS scores, there was no main effect for CLD group (p=0.466), and the scores did not change significantly over time (p=0.548; online supplemental figure S4). The least squares means for WI-NRS were approximately within one point for each CLD, with the most variation in MASH, and across all survey time points. Although the covariates for CLD group and study time point were not significant, the interaction term between the two covariates was significant (p=0.022), indicating a cross-over interaction where the group mean that was the highest at baseline (ie, the WI-NRS scores reported by participants with PSC) dropped below the means reported by other groups (ie, participants with MASH and PBC) at the month 3 time point. Given the lack of substantial change in WI-NRS scores during the observation period, this significant result should be interpreted with caution.

## Discussion

We present findings from a longitudinal, survey-based cohort of participants with CLD and highlight that pruritus is common, persistent and burdensome for patients irrespective of aetiology. Based on WI-NRS with a 2-week recall, 49%–65% of screened CLD cohorts with over 50 participants had moderate-to-severe pruritus. While more common among individuals with chronic biliary disease, moderate-to-severe pruritus was also reported by nearly 50% of screened participants with MASH. Although this frequency is higher than previous evidence suggests, it is possible that some patients are not aware that pruritus could relate to underlying liver disease and do not report it to their doctor. We have previously shown that the frequency of pruritus documented in the medical records of patients with PBC is significantly lower than that self-reported by patients.^25^ Indeed, approximately one in four participants in this study reported not being asked about pruritus by their HCP. It is also possible that this finding is due to self-selection bias.

The effects of pruritus on patients’ lives are also demonstrated by mean 5-D itch scores, where the highest (worst) scores among participants with severe pruritus on the WI-NRS, particularly on the disability domain, demonstrate the impact pruritus has on day-to-day activities, including sleep, social life and work. These results are similar to the TARGET-PBC cohort, where patients with clinically significant pruritus (according to the PBC-40 itch domain) scored significantly worse than those with mild itch across all 5-D itch scale domains.^4^ Our study also highlights the burden of pruritus over time, describing patients’ journey with pruritus longitudinally. Overall, the mean number of months since the onset of pruritus to enrolment ranged from 28.0 to 77.5 months, indicating that pruritus is a chronic problem for many patients; this is supported by the prospective analysis, which demonstrates the persistence of pruritus over 6 months. The least squares means for WI-NRS were approximately within one point for each CLD, with the most variation in MASH and across all survey time points. This very small magnitude of change should be interpreted with extreme caution, as it is not clinically meaningful without additional data.^26 27^ Furthermore, at baseline, approximately 69%–83% of enrolled participants reported that the frequency and severity of pruritus was either the same or worse compared with when it first began. For the 5-D itch scale, participants across CLDs reported a score of 3–<4 for the direction domain, implying that pruritus was slightly better but still present or unchanged since onset, despite currently available anti-itch therapies. Approximately one-third of enrolled participants across cohorts experienced pruritus every day.

The SF-36v2 health survey indicates deficits in HRQoL in each of the CLDs studied. With few exceptions, the differences in mean SF-36v2 scale and summary measure scores from the norm were in the moderate-to-large effect size range and exceeded thresholds of clinically meaningful differences compared with the general population.^28^ Across CLDs, the SF-36v2 general health domain showed the greatest deficits; this is likely attributable to the presence of a chronic disease and its impact on future health outlook. In addition, significant deficits were identified in role functioning, as measured by physical and emotional scales, social functioning and vitality domains.

While effect size characterisation does not address the clinical meaningfulness of HRQoL burden, the SF-36v2 scores observed at baseline are similar to those in other chronic diseases with well-known morbidity, including diabetes, rheumatoid arthritis and osteoarthritis.^28^ The incremental impact of pruritus on QoL has been described previously; in one study, in patients with HCV and pruritus, it was found that each unit increase in NRS was associated with a 2–3-unit decline in reported QoL for emotional well-being, general health, physical function, energy/fatigue, social functioning and the impact of emotional health on QoL.^29^

Although overall scores between CLDs did not differ dramatically, within each CLD, greater pruritus severity was associated with worse SF-36v2 domains scores. This highlights the increased health, social and physical burden of pruritus on patients with increased pruritus severity.

The most common descriptors of pruritus, such as ‘deep itch’ and ‘urgent itch’, were similar across CLDs, indicating a common experience. Additionally, many participants, especially those with PBC and PSC, associated their pruritus with the terms ‘relentless’, “I want to tear my skin off” and “itch until I bleed”, illustrating the severity of this condition that is generally not relieved by scratching. Incidentally, a range of 30% (HCV) to 51% (PBC) of participants across cohorts reported that scratching does not help. These findings may be useful in developing instruments to screen patients who may have pruritus but do not know how it links to their CLD, or as the basis for PRO measures specific to the experience of itch. For the PBC, PSC and HCV groups, most participants reported that their HCP talked to them about their pruritus. However, 5%–23% of participants reported that their HCP had not spoken with them about their pruritus. Reciprocally, patients with CLDs may not associate their pruritus with their CLD, subsequently not raising it to their HCPs, resulting in pruritus being overlooked.

In terms of treatment strategies, most enrolled participants used topical remedies and antihistamines. Up to 16% of participants across CLDs reported no improvement in pruritus when using topical remedies, and up to 23% reported the same response for antihistamines, supported by the lack of a definitive evidence base.^4 30^ This may reflect the gaps in knowledge, limited treatment guidelines and clinical trial data on treatments for pruritus in CLD^4 10^ or the fact that current treatments for pruritus are perceived to be largely ineffective or poorly tolerated.^34 10 25 31^,^36^ A prospective and systematic evaluation of pruritus onset and its occurrence in line with CLD severity and clinically relevant variables is needed, alongside parallel studies of current and newly available anti-pruritus agents.

We acknowledge several limitations. Participants were predominantly recruited via patient groups and panels. Despite a deliberate attempt to mask the purpose of the study from potential participants, the individuals coming forward may have reflected a self-selected population more motivated to participate in research or those predisposed to selection or reporting biases, such as those with more severe and burdensome disease or those with an interest in HRQoL. For example, participants interested in this study may be more likely to report their symptoms and their impacts on QoL. This may explain the higher prevalence of pruritus in HCV and female preponderance of the recruited PSC group, which are not aligned with the known disease epidemiology. Due to the predominant recruitment via patient groups, the aim of the study may have been inadvertently leaked or disseminated; however, the risk of this is believed to be low, as participants and referring clinicians had no knowledge of the pruritus-related objectives. Age, socio-economic status and computer literacy may restrict the generalisability of results since participants required access to a device (eg, computer, tablet) and internet connectivity to complete the surveys. Participants were recruited from Western countries, further limiting the generalisability of the findings. Data were not adjusted to address the potential non-representativeness of the study sample. Additionally, as all information collected for this study was self-reported, these data may have been prone to response and recall biases. As an example, the presence of pruritus at enrolment might be particularly sensitive to recall bias given the relatively long timeframe (3 months) for recall. In addition, estimates of pruritus frequency among patients with viral hepatitis are higher than those previously reported.^17 29^ A relatively large proportion of those screened did not meet the criteria for enrolment, meaning that the included population may not reflect the general HBV and HCV populations.

Perhaps most importantly, confirmation of CLD did not include review of clinical imaging, laboratory test results or individual patient medical records by any of the investigators to ascertain stage or severity. Thus, it was not possible to determine whether and how pruritus intensity associated with liver disease extent or severity or how well individual cohorts reflect real-world CLD cohorts. The statistical tests conducted to understand pruritus characteristics did not adjust for potential confounders. As our study was hypothesis-generating, potential confounders were not clear. Finally, although HRQoL burden was significant, the impact of liver disease activity, stage and existing responses to therapy could not be separated from the impact of pruritus. Nevertheless, the HRQoL impact stratified by pruritus severity suggests that pruritus has an incremental impact.

In summary, initial findings from this study highlight that pruritus in general (as well as moderate-to-severe pruritus specifically) is common, persistent and can have a profound impact on patients with CLDs over time. As this study is limited by potential selection and recall bias due to the method of patient recruitment and reporting, further research is required to understand the drivers of pruritus occurrence in patients, the therapeutic shortfall in an era of current anti-itch treatments and factors influencing persistence versus resolution of symptoms over time to increase generalisability.

## Supplementary material

10.1136/bmjgast-2025-001809 online supplemental file 1

10.1136/bmjgast-2025-001809 online supplemental file 2

10.1136/bmjgast-2025-001809 online supplemental file 3

## Data Availability

Data are available on reasonable request. All data relevant to the study are included in the article or uploaded as supplementary information.

## References

[1] de Veer RC, van Hooff MC, da Silva G (2023). Quality of life in Dutch patients with primary biliary cholangitis: Discrepancies between patients’ perspectives and objective disease parameters. Hepatol Res.

[2] Dean R, Yazdanfar M, Zepeda J (2023). Investigating the cholestatic pruritus of primary sclerosing cholangitis (ItCh-PSC): a study of patients participating in the consortium for autoimmune liver disease (CALiD). J Hepatol.

[3] Hegade VS, Mells GF, Fisher H (2019). Pruritus Is Common and Undertreated in Patients With Primary Biliary Cholangitis in the United Kingdom. Clin Gastroenterol Hepatol.

[4] Mayo MJ, Carey E, Smith HT (2023). Impact of Pruritus on Quality of Life and Current Treatment Patterns in Patients with Primary Biliary Cholangitis. Dig Dis Sci.

[5] Hussain N, Ferguson J, Abbas N (2024). THU-160 Long-term variability of pruritus in primary sclerosing cholangitis and implications for future clinical trial design. J Hepatol.

[6] Gungabissoon U, Smith HT, von Maltzahn R (2024). Pruritus in primary biliary cholangitis is under-recorded in patient medical records. BMJ Open Gastroenterol.

[7] Hussain N, Hirschfield B, Ferguson J (2023). P34 Burden, impact and variability of pruritus in primary sclerosing cholangitis (psc) over time: a prospective observational study. Gut.

[8] Mells GF, Pells G, Newton JL (2013). Impact of primary biliary cirrhosis on perceived quality of life: the UK-PBC national study. Hepatology.

[9] Rishe E, Azarm A, Bergasa NV (2008). Itch in primary biliary cirrhosis: a patients’ perspective. Acta Derm Venereol.

[10] Tajiri K, Shimizu Y (2017). Recent advances in the management of pruritus in chronic liver diseases. World J Gastroenterol.

[11] Montagnese S, Nsemi LM, Cazzagon N (2013). Sleep-Wake profiles in patients with primary biliary cirrhosis. *Liver Int*.

[12] PSC Support (2016). Clinical need in PSC and clinically meaningful change: what is important to patients?. https://www.pscsupport.org.uk/wp-content/uploads/2019/06/PSC-Support-Patient-Survey-Results.pdf.

[13] Walmsley M, Leburgue A, Thorburn D (2019). FRI-062-Identifying research priorities in primary sclerosing cholangitis: Driving clinically meaningful change from the patients’ perspective. J Hepatol.

[14] Gungabissoon U, Smith HT, von Maltzahn R (2022). Characterizing patients with pruritus and primary biliary cholangitis (PBC) in the PicnicHealthPBC cohort; contrasting medical record-documented pruritus with patient-reported pruritus from the PBC-40 (Poster No. 4748). AASLD.

[15] Abbas N, Smith R, Flack S (2024). Critical shortfalls in the management of PBC: Results of a UK-wide, population-based evaluation of care delivery. *JHEP Rep*.

[16] Ahmadi AR, Chicco M, van den Berge M (2020). Rifampicin for Treatment of Cholestatic Pruritus Caused by Drug-Induced Acute Liver Injury as Assessed by the RUCAM Classification. Case Reports Hepatol.

[17] Oeda S, Takahashi H, Yoshida H (2018). Prevalence of pruritus in patients with chronic liver disease: A multicenter study. Hepatol Res.

[18] Younossi ZM, Wong VW-S, Anstee QM (2020). Fatigue and Pruritus in Patients with Advanced Fibrosis Due to Nonalcoholic Steatohepatitis: The Impact on Patient-Reported Outcomes. *Hepatol Commun*.

[19] Cook NS, Nagar SH, Jain A (2019). Understanding Patient Preferences and Unmet Needs in Non-alcoholic Steatohepatitis (NASH): Insights from a Qualitative Online Bulletin Board Study. Adv Ther.

[20] Younossi ZM, Guyatt G, Kiwi M (1999). Development of a disease specific questionnaire to measure health related quality of life in patients with chronic liver disease. Gut.

[21] Yoshikawa S, Asano T, Morino M (2021). Pruritus is common in patients with chronic liver disease and is improved by nalfurafine hydrochloride. Sci Rep.

[22] Xu J, Wang Y, Khoshdeli M (2023). IL-31 levels correlate with pruritus in patients with cholestatic and metabolic liver diseases and is farnesoid X receptor responsive in NASH. Hepatology.

[23] Confirmit (2018). Confirmit horizons v24.

[24] Reich A, Chatzigeorkidis E, Zeidler C (2017). Tailoring the Cut-off Values of the Visual Analogue Scale and Numeric Rating Scale in Itch Assessment. Acta Derm Venerol.

[25] Gungabissoon U, Gibbons DC, Requena G (2022). Disease burden of primary biliary cholangitis and associated pruritus based on a cross-sectional US claims analysis. BMJ Open Gastroenterol.

[26] Levy C, Kremer AE, Skalicky A (2025). THU-291 Clinical meaningful change of pruritus numeric rating scale in adults with primary biliary cholangitis with moderate-to-severe pruritus. J Hepatol.

[27] Vernon MK, Swett LL, Speck RM (2021). Psychometric validation and meaningful change thresholds of the Worst Itching Intensity Numerical Rating Scale for assessing itch in patients with chronic kidney disease-associated pruritus. J Patient Rep Outcomes.

[28] Maruish ME (2011). User's manual for the SF-36v2 health survey: Quality Metric Incorporated.

[29] Lu M, Rupp LB, Melkonian C (2024). Persistent pruritus associated with worse quality of life in patients with chronic hepatitis. *Liver Int*.

[30] Song J, Xian D, Yang L (2018). Pruritus: Progress toward Pathogenesis and Treatment. Biomed Res Int.

[31] Electronic Medicines Compendium (2022). Questran light 4g/sachet powder for oral suspension. https://www.medicines.org.uk/emc/product/10588/smpc.

[32] Hirschfield GM, Beuers U, Corpechot C (2017). EASL Clinical Practice Guidelines: The diagnosis and management of patients with primary biliary cholangitis. J Hepatol.

[33] FDA (2010). Rifadin. https://www.accessdata.fda.gov/drugsatfda_docs/label/2010/050420s073,050627s012lbl.pdf.

[34] Lindor KD, Bowlus CL, Boyer J (2019). Primary Biliary Cholangitis: 2018 Practice Guidance from the American Association for the Study of Liver Diseases. Hepatology.

[35] Talwalkar JA, Lindor KD (2003). Primary biliary cirrhosis. Lancet.

[36] Yosipovitch G, Bernhard JD (2013). Clinical practice. Chronic Pruritus. N Engl J Med.

[37] Elman S, Hynan LS, Gabriel V (2010). The 5-D itch scale: a new measure of pruritus. Br J Dermatol.

