# Supplementary material for: Pruritus and health-related quality of life in chronic liver disease: a longitudinal, survey-based cohort study
Source: BMJ Open Gastroenterol. 2025 Dec 11;12(1):e001809. doi: 10.1136/bmjgast-2025-001809 (PMC12699617; doi:10.1136/bmjgast-2025-001809)
Supplement: online supplemental file 1 [file bmjgast-12-1-s001.docx]

Supplementary materials

## Supplementary Tables and Figures

### Table S1. Baseline demographics of patients with AIH, HBV and DILI, and all enrolled patients (all CLDs)

| **Characteristic** | **AIH**  **(n=36)** | **HBV**  **(n=28)** | **DILI**  **(n=19)** | **Total**  **(N=357)^a^** |
| --- | --- | --- | --- | --- |
| **Age, years** |  |  |  |  |
| **Mean (SD)** | 49.8 (14.6) | 44.1 (11.9) | 51.1 (12.0) | 51.3 (12.8) |
| Median (IQR) | 51.0 (23.0) | 42.5 (16.5) | 53.0 (24.0) | 52.0 (19.0) |
| **Time since chronic liver disease diagnosis, years^b^** |  |  |  |  |
| Mean (SD) | 6.8 (7.8) | 14.0 (9.4) | 3.8 (2.8) | 6.9 (6.8) |
| Median (IQR) | 4.0 (5.5) | 12.5 (11.5) | 3.0 (5.0) | 5.0 (7.0) |
| **Sex, n (%)** |  |  |  |  |
| Female | 26 (72.2) | 11 (39.3) | 10 (52.6) | 227 (63.6) |
| Male | 10 (27.8) | 17 (60.7) | 9 (47.4) | 130 (36.4) |
| **Country, n (%)** |  |  |  |  |
| UK | 11 (30.6) | 2 (7.1) | 0 | 69 (19.3) |
| US | 6 (16.7) | 7 (25.0) | 6 (31.6) | 149 (41.7) |
| Canada | 6 (16.7) | 0 | 0 | 31 (8.7) |
| Germany | 13 (36.1) | 19 (67.9) | 13 (68.4) | 108 (30.3) |

Tables are ordered by the prevalence in the left-most column. Due to rounding, percentages may not add to 100%.

^a^The proportion of patients with ALD or MASLD experiencing pruritus was not presented due to the limited size of these screened populations (N=1 and 4 for ALD and MASLD, respectively, at baseline). ^b^At screening, approximate year of CLD diagnosis was captured. Time since diagnosis was calculated as the difference (in years) between screening year and year of diagnosis.

AIH, autoimmune hepatitis; ALD, alcoholic liver disease; CLD, chronic liver disease; DILI, drug-induced liver injury; HBV, chronic hepatitis B virus infection; IQR, interquartile range; MASLD, metabolic dysfunction-associated steatotic liver disease; SD, standard deviation; UK, United Kingdom; US, United States.

### Table S2. Participant experience of PBC, PSC, MASH, and HCV at baseline

| **Characteristic** | **PBC**  **(N=80)** | **PSC**  **(N=74)** | **MASH**  **(N=61)** | **HCV**  **(N=54)** |
| --- | --- | --- | --- | --- |
| **In the time since your itch/pruritus began, how would you describe the sensation of your itch/pruritus? Select all that apply (n [%])** |  |  |  |  |
| Deep itch | 54 (67.5) | 57 (77.0) | 39 (63.9) | 29 (53.7) |
| Urgent itch | 46 (57.5) | 45 (60.8) | 36 (59.0) | 34 (63.0) |
| Scratching does not help | 41 (51.3) | 33 (44.6) | 28 (45.9) | 16 (29.6) |
| Relentless | 36 (45.0) | 38 (51.4) | 27 (44.3) | 21 (38.9) |
| Itch until I bleed | 36 (45.0) | 38 (51.4) | 13 (21.3) | 10 (18.5) |
| Bugs crawling | 34 (42.5) | 23 (31.1) | 18 (29.5) | 13 (24.1) |
| Want to tear my skin off | 33 (41.3) | 31 (41.9) | 19 (31.1) | 13 (24.1) |
| Prickly/ needles | 33 (41.3) | 26 (35.1) | 20 (32.8) | 15 (27.8) |
| Burning | 33 (41.3) | 18 (24.3) | 22 (36.1) | 23 (42.6) |
| Hives | 11 (13.8) | 9 (12.2) | 5 (8.2) | 4 (7.4) |
| Itch feels like it is “in the veins” | 13 (16.3) | 16 (21.6) | 3 (4.9) | 5 (9.3) |
| Other | 4 (5.0) | 6 (8.1) | 1 (1.6) | 0 (0.0) |
| **In the time since your itch/pruritus began, in general, at what time of day has your itch/pruritus been the worst? (n [%])** |  |  |  |  |
| During the night | 40 (50.0) | 43 (58.1) | 16 (26.2) | 8 (14.8) |
| About the same during the day and night | 21 (26.3) | 15 (20.3) | 25 (41.0) | 17 (31.5) |
| During the day | 19 (23.8) | 16 (21.6) | 20 (32.8) | 29 (53.7) |
| **In the time since your itch/pruritus began, during which season is your itch/pruritus worst? Select all that apply (n [%])** |  |  |  |  |
| Spring | 1 (1.3) | 7 (9.5) | 5 (8.2) | 4 (7.4) |
| Summer | 8 (10.0) | 14 (18.9) | 13 (21.3) | 8 (14.8) |
| Fall/Autumn | 4 (5.0) | 3 (4.1) | 1 (1.6) | 2 (3.7) |
| Winter | 8 (10.0) | 8 (10.8) | 5 (8.2) | 5 (9.3) |
| Don’t know | 14 (17.5) | 8 (10.8) | 11 (18.0) | 9 (16.7) |
| My itch/pruritus is the same across seasons | 49 (61.3) | 44 (59.5) | 31 (50.8) | 31 (57.4) |

Tables are ordered from highest to lowest response frequency in participants with PBC, excluding seasonality.HCV, chronic hepatitis C virus infection; MASH, metabolic dysfunction-associated steatohepatitis; PBC, primary biliary cholangitis; PSC, primary sclerosing cholangitis.

### Table S3. Pruritus management among participants with PBC, PSC, MASH, and HCV at baseline

| **Characteristic** | **PBC**  **(N=80)** | **PSC**  **(N=74)** | **MASH**  **(N=61)** | **HCV**  **(N=54)** |
| --- | --- | --- | --- | --- |
| **In the time since your itch/pruritus began, how has your healthcare provider approached your itch/pruritus? Select all that apply (n [%])** |  |  |  |  |
| Talked to me about my itch/pruritus | 48 (60.0) | 56 (75.7) | 37 (60.7) | 39 (72.2) |
| Prescribed medication for my itch/pruritus | 36 (45.0) | 45 (60.8) | 27 (44.3) | 25 (46.3) |
| Looked at my skin | 21 (26.3) | 28 (37.8) | 38 (62.3) | 33 (61.1) |
| Has not talked to me about my itch/pruritus | 17 (21.3) | 4 (5.4) | 14 (23.0) | 9 (16.7) |
| Other | 10 (12.5) | 7 (9.5) | 6 (9.8) | 2 (3.7) |
| Gave me questionnaires about my itch/pruritus | 8 (10.0) | 9 (12.2) | 4 (6.6) | 9 (16.7) |
| Performed biopsy of my skin / took skin sample | 3 (3.8) | 10 (13.5) | 3 (4.9) | 5 (9.3) |
| **At any time since your itch/pruritus began, have you used any of the following for your itch/pruritus? Select all that apply (n [%])** |  |  |  |  |
| Topical remedies | 59 (73.8) | 61 (82.4) | 45 (73.8) | 44 (81.5) |
| Antihistamines | 42 (52.5) | 54 (73.0) | 34 (55.7) | 21 (38.9) |
| Bile acid sequestrants / Anion-exchange resins | 23 (28.8) | 36 (48.6) | 2 (3.3) | 1 (1.9) |
| Gabapentin | 16 (20.0) | 7 (9.5) | 12 (19.7) | 9 (16.7) |
| Selective serotonin reuptake inhibitors | 10 (12.5) | 12 (16.2) | 9 (14.8) | 8 (14.8) |
| Light therapy | 9 (11.3) | 11 (14.9) | 3 (4.9) | 3 (5.6) |
| Other | 9 (11.3) | 9 (12.2) | 3 (4.9) | 5 (9.3) |
| Rifampicin/rifampin | 7 (8.8) | 9 (12.2) | 1 (1.6) | 0 |
| Oral opiate antagonists | 6 (7.5) | 8 (10.8) | 2 (3.3) | 2 (3.7) |
| Plasmapheresis | 0 | 1 (1.4) | 0 | 1 (1.9) |
| Albumin dialysis | 0 | 0 | 0 | 1 (1.9) |
| Phenobarbital | 0 | 0 | 0 | 1 (1.9) |
| Molecular adsorbent recirculating system | 0 | 1 (1.4) | 0 | 0 |
| Nasobiliary or external biliary drainage | 0 | 1 (1.4) | 0 | 0 |
| No treatment | 10 (12.5) | 4 (5.4) | 6 (9.8) | 6 (11.1) |
| **Are you currently using any of the following for your itch/pruritus? Select all that apply (n [%])** |  |  |  |  |
| Topical remedies | 43 (53.8) | 44 (59.5) | 40 (65.6) | 41 (75.9) |
| Antihistamines | 23 (28.8) | 29 (39.2) | 26 (42.6) | 16 (29.6) |
| Bile acid sequestrants / Anion-exchange resins | 11 (13.8) | 20 (27.0) | 0 | 1 (1.9) |
| Selective serotonin reuptake inhibitors | 6 (7.5) | 7 (9.5) | 9 (14.8) | 6 (11.1) |
| Other | 5 (6.3) | 6 (8.1) | 2 (3.3) | 4 (7.4) |
| Gabapentin | 5 (6.3) | 4 (5.4) | 9 (14.8) | 7 (13.0) |
| Light therapy | 4 (5.0) | 5 (6.8) | 1 (1.6) | 2 (3.7) |
| Oral opiate antagonists | 3 (3.8) | 4 (5.4) | 1 (1.6) | 1 (1.9) |
| Rifampicin/rifampin | 2 (2.5) | 6 (8.1) | 0 | 0 |
| Plasmapheresis | 0 | 1 (1.4) | 0 | 0 |
| Phenobarbital | 0 | 0 | 0 | 1 (1.9) |
| Albumin dialysis | 0 | 0 | 0 | 0 |
| Molecular adsorbent recirculating system | 0 | 0 | 0 | 0 |
| Nasobiliary or external biliary drainage | 0 | 0 | 0 | 0 |
| No treatment^a^ | 14 (17.5) | 15 (20.3) | 4 (6.6) | 3 (5.6) |
| **In the time since your itch/pruritus began, please indicate how much each of the following improves your itch/pruritus (n [%])** |  |  |  |  |
| Something cool on the skin |  |  |  |  |
| Have not tried | 18 (22.5) | 12 (16.2) | 12 (19.7) | 8 (14.8) |
| Not at all | 10 (12.5) | 6 (8.1) | 2 (3.3) | 5 (9.3) |
| Slightly improved | 29 (36.3) | 21 (28.4) | 18 (29.5) | 15 (27.8) |
| Somewhat improved | 14 (17.5) | 15 (20.3) | 11 (18.0) | 20 (37.0) |
| Much improved | 7 (8.8) | 12 (16.2) | 12 (19.7) | 6 (11.1) |
| Very much improved | 2 (2.5) | 8 (10.8) | 6 (9.8) | 0 |
| Over-the-counter topical creams, moisturisers, etc. |  |  |  |  |
| Have not tried | 10 (12.5) | 12 (16.2) | 3 (4.9) | 4 (7.4) |
| Not at all | 13 (16.3) | 11 (14.9) | 6 (9.8) | 3 (5.6) |
| Slightly improved | 30 (37.5) | 25 (33.8) | 15 (24.6) | 15 (27.8) |
| Somewhat improved | 21 (26.3) | 20 (27.0) | 19 (31.1) | 17 (31.5) |
| Much improved | 6 (7.5) | 4 (5.4) | 9 (14.8) | 11 (20.4) |
| Very much improved | 0 | 2 (2.7) | 9 (14.8) | 4 (7.4) |
| Prescription medications |  |  |  |  |
| Have not tried | 36 (45.0) | 22 (29.7) | 33 (54.1) | 26 (48.1) |
| Not at all | 7 (8.8) | 6 (8.1) | 1 (1.6) | 3 (5.6) |
| Slightly improved | 10 (12.5) | 9 (12.2) | 6 (9.8) | 3 (5.6) |
| Somewhat improved | 10 (12.5) | 19 (25.7) | 8 (13.1) | 10 (18.5) |
| Much improved | 12 (15.0) | 14 (18.9) | 5 (8.2) | 3 (5.6) |
| Very much improved | 5 (6.3) | 4 (5.4) | 8 (13.1) | 9 (16.7) |
| Over-the-counter antihistamines |  |  |  |  |
| Have not tried | 37 (46.3) | 25 (33.8) | 25 (41.0) | 31 (57.4) |
| Not at all | 17 (21.3) | 17 (23.0) | 4 (6.6) | 4 (7.4) |
| Slightly improved | 10 (12.5) | 14 (18.9) | 11 (18.0) | 7 (13.0) |
| Somewhat improved | 12 (15.0) | 16 (21.6) | 10 (16.4) | 6 (11.1) |
| Much improved | 3 (3.8) | 2 (2.7) | 7 (11.5) | 4 (7.4) |
| Very much improved | 1 (1.3) | 0 | 4 (6.6) | 2 (3.7) |
| Scratching |  |  |  |  |
| Have not tried | 1 (1.3) | 0 | 2 (3.3) | 1 (1.9) |
| Not at all | 39 (48.8) | 26 (35.1) | 30 (49.2) | 19 (35.2) |
| Slightly improved | 22 (27.5) | 26 (35.1) | 18 (29.5) | 21 (38.9) |
| Somewhat improved | 12 (15.0) | 15 (20.3) | 8 (13.1) | 8 (14.8) |
| Much improved | 5 (6.3) | 4 (5.4) | 2 (3.3) | 3 (5.6) |
| Very much improved | 1 (1.3) | 3 (4.1) | 1 (1.6) | 2 (3.7) |

Tables are ordered from highest to lowest response frequency in participants with PBC, excluding ordinal variables and “No treatment” category.
^a^Item was only asked to participants who indicated at least one treatment ever.
HCV, chronic hepatitis C virus infection; MASH, metabolic dysfunction-associated steatohepatitis; PBC, primary biliary cholangitis; PSC, primary sclerosing cholangitis.

### Table S4. Adjustments to lifestyle due to pruritus among participants with PBC, PSC, MASH, and HCV at baseline

| **Characteristic** | **PBC**  **(N=80)** | **PSC**  **(N=74)** | **MASH**  **(N=61)** | **HCV**  **(N=54)** |
| --- | --- | --- | --- | --- |
| **In the time since your itch/pruritus began, please select any adjustments to your lifestyle that you had to make due to your itch/pruritus (n [%]) Select all that apply (n [%])** |  |  |  |  |
| Changed choices in fashion or clothing | 35 (43.8) | 30 (40.5) | 24 (39.3) | 18 (33.3) |
| Changed diet | 29 (36.3) | 23 (31.1) | 19 (31.1) | 18 (33.3) |
| Changed alcohol drinking habits | 22 (27.5) | 21 (28.4) | 7 (11.5) | 11 (20.4) |
| Spent less time on hobbies or leisure time activities | 21 (26.3) | 12 (16.2) | 8 (13.1) | 10 (18.5) |
| Changed home climate control systems | 15 (18.8) | 18 (24.3) | 15 (24.6) | 14 (25.9) |
| Other | 14 (17.5) | 17 (23.0) | 3 (4.9) | 3 (5.6) |
| Stopped working altogether | 12 (15.0) | 9 (12.2) | 4 (6.6) | 11 (20.4) |
| Changed jobs or required some sort of adjustment/change in the workplace | 5 (6.3) | 11 (14.9) | 2 (3.3) | 2 (3.7) |
| Were unable to do daily chores/tasks | 5 (6.3) | 6 (8.1) | 3 (4.9) | 3 (5.6) |
| Changed vacation plans to find more favourable weather | 3 (3.8) | 5 (6.8) | 3 (4.9) | 2 (3.7) |
| Moved to find a more favourable climate | 1 (1.3) | 1 (1.4) | 1 (1.6) | 2 (3.7) |
| None | 23 (28.8) | 19 (25.7) | 19 (31.1) | 16 (29.6) |

Tables are ordered from highest to lowest response frequency in participants with PBC, excluding the “None” category.
HCV, chronic hepatitis C virus infection; MASH, metabolic dysfunction-associated steatohepatitis; PBC, primary biliary cholangitis; PSC, primary sclerosing cholangitis.

### Figure S1. Participant disposition

^
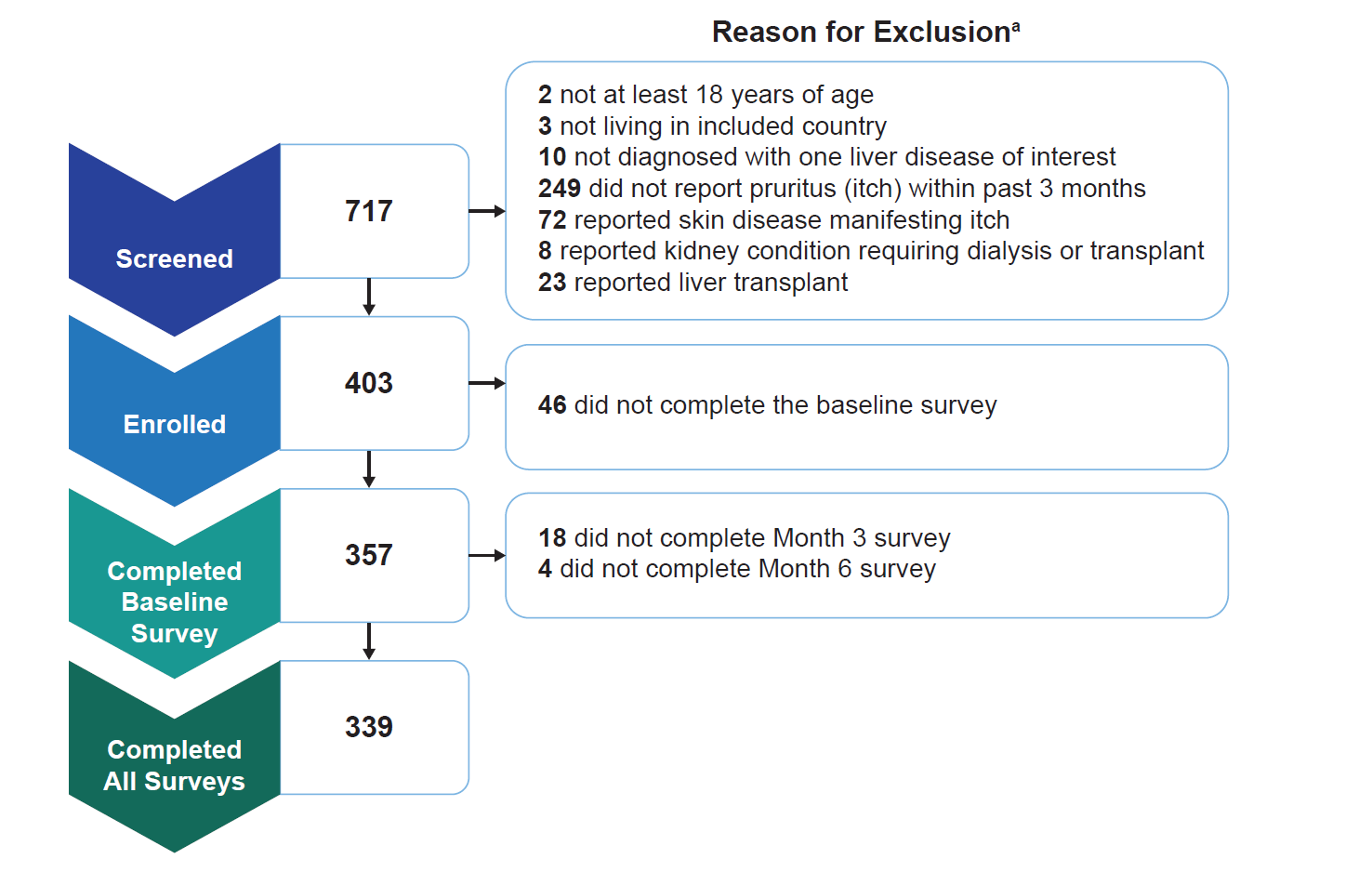
^

^a^Reasons for exclusion are not mutually exclusive.

### Figure S2. Baseline 5-D itch scale mean domain and total scores by pruritus severity in participants with (A) PBC, (B) PSC, (C) MASH, and (D) HCV


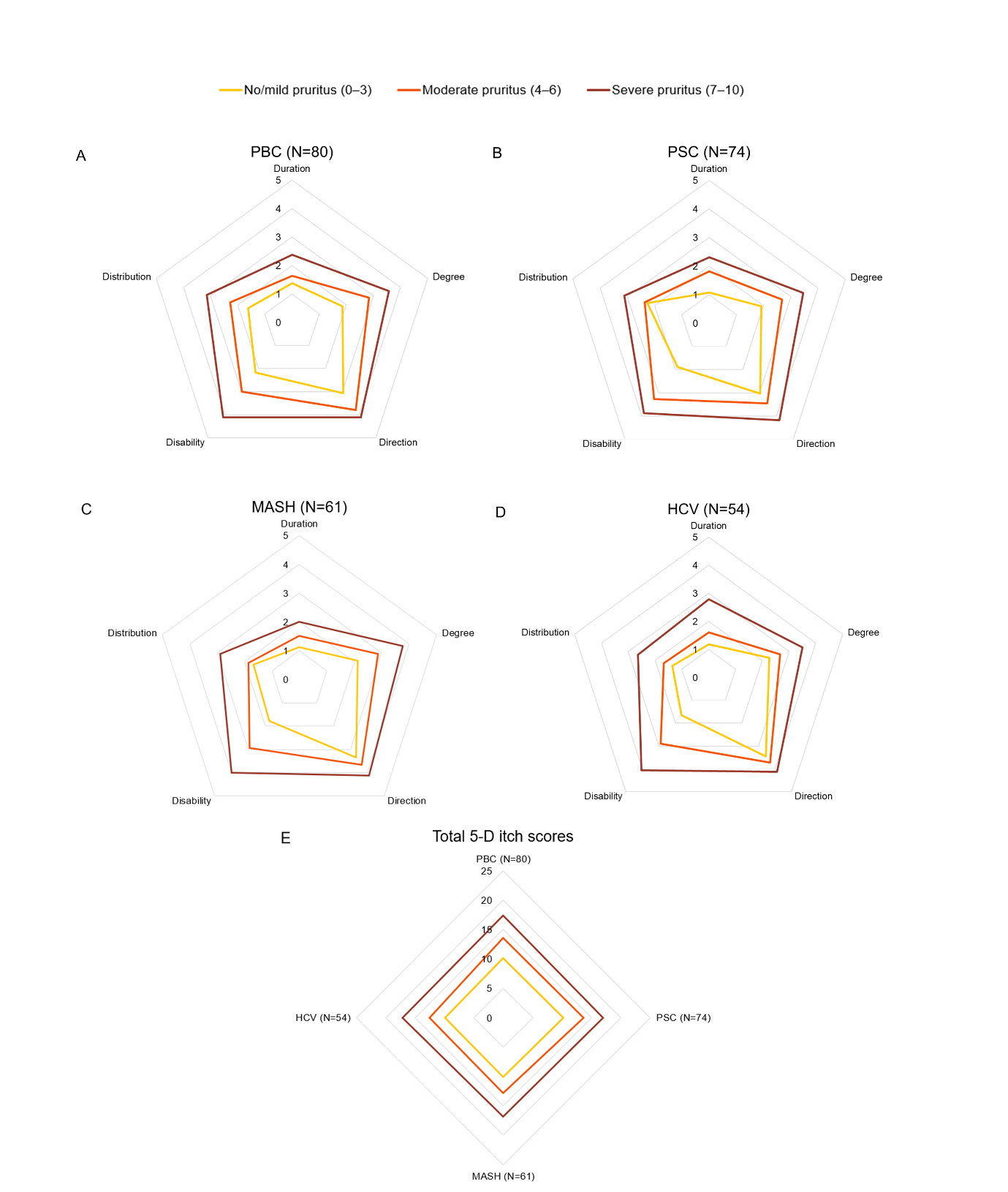


Pruritus severity was determined by WI-NRS: No/mild itch, 0–3; Moderate itch, 4–6; Severe itch, 7–10. Higher 5-D itch scale scores indicate higher pruritus severity.

5-D, five dimension; HCV, chronic hepatitis C virus infection; MASH, metabolic dysfunction-associated steatohepatitis; PBC, primary biliary cholangitis; PSC, primary sclerosing cholangitis; WI-NRS, worst-itch numerical rating scale.

### Figure S3. Baseline SF-36v2 mean scores by pruritus severity in participants with PBC, PSC, MASH, and HCV


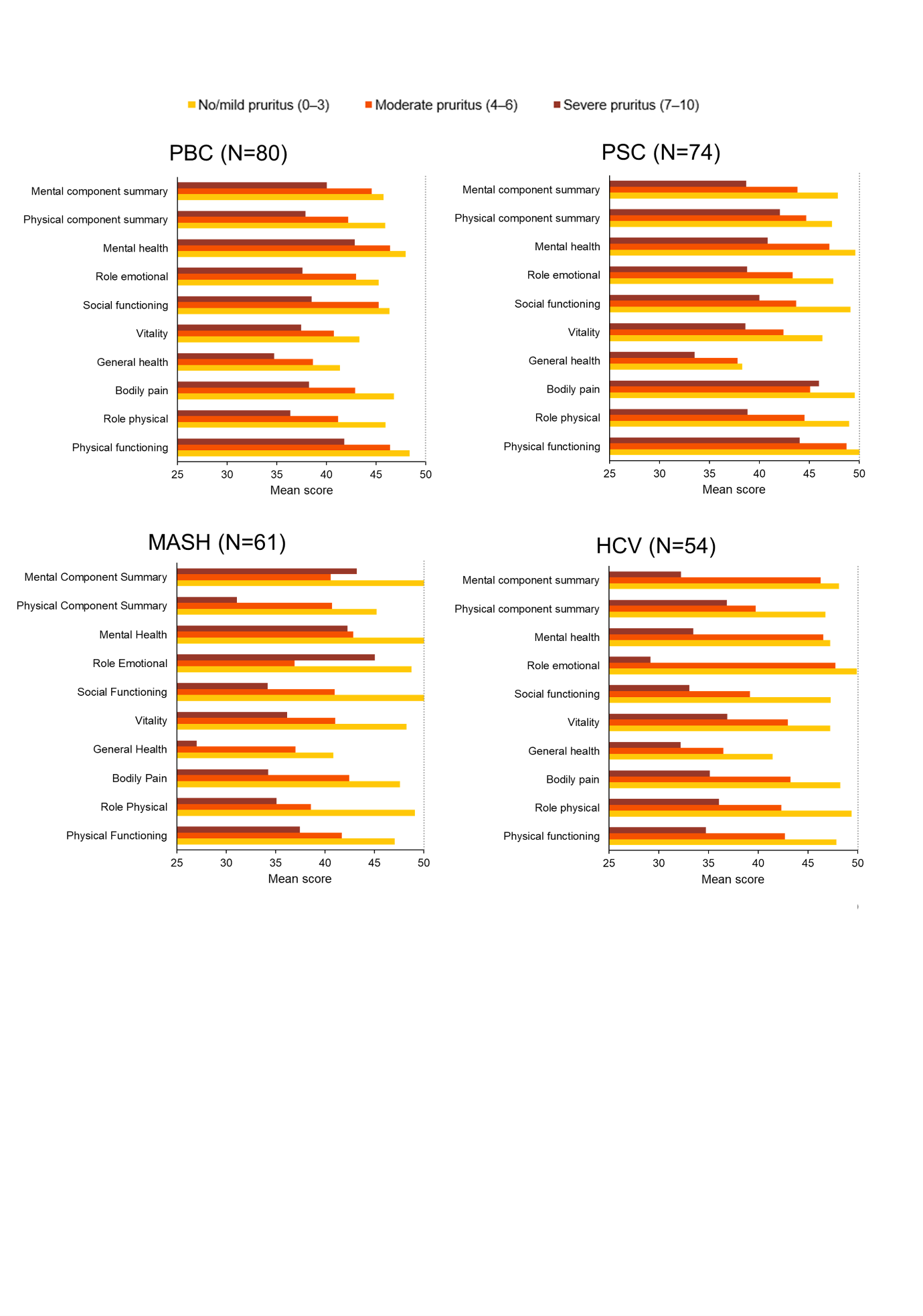


The grey line indicates the general population norm. Pruritus severity was determined by WI-NRS: No/mild itch, 0–3; Moderate itch, 4–6; Severe itch, 7–10. Higher SF-36v2 scores indicate better health-related quality of life.

HCV, chronic hepatitis C virus infection; MASH, metabolic dysfunction-associated steatohepatitis; PBC, primary biliary cholangitis; PSC, primary sclerosing cholangitis; SF-36v2, 36-Item Short-Form Survey; WI-NRS, worst-itch numerical rating scale.

### Figure S4. Least square means for the WI-NRS rating by timepoint and CLD

**
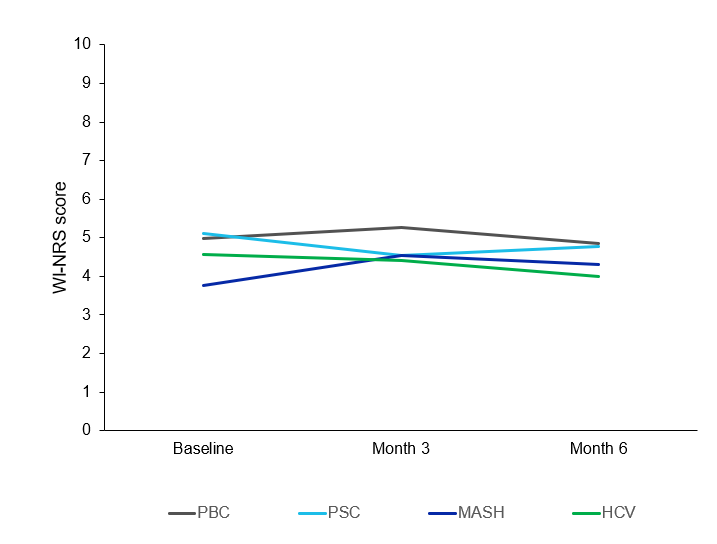
**

Least square means were obtained from a linear repeated-measures mixed model where the WI-NRS score was included as the independent variable. The model includes fixed effects for age at baseline, gender, time since diagnosis (assessed at baseline), liver disease (p=0.466), and study timepoint (p= 0.548). Each model included an interaction term between study timepoint and liver disease (p=0.022). WI-NRS scores indicate the following: No/mild pruritus, 0–3; Moderate pruritus, 4–6; Severe pruritus, 7–10.

CLD, chronic liver disease; HCV, chronic hepatitis C virus infection; MASH, metabolic dysfunction-associated steatohepatitis; PBC, primary biliary cholangitis; PSC, primary sclerosing cholangitis; WI-NRS, worst-itch numerical rating scale.

## Supplementary Methods

### Recruitment language

Recruitment emails and social media posts used the subject line “Online Survey Research Opportunity: Study on Liver Disease” and included the aim “The purpose of this study is to understand the experience of patients with liver disease, including symptoms and impact on quality of life.” Potential participants were sent recruitment messages that contained a link to an online screener. Screening questions included questions about demographics, chronic liver disease (CLD; e.g., year of diagnosis or the type of CLD), and comorbidities, as well as pruritus. Measures were implemented to avoid duplicate participants, including checks for duplicate participant information and blocking duplicate IP addresses.

### Exclusion criteria

Participants were excluded if they had any of the following: a diagnosis of more than one of the aforementioned target CLDs (participants with autoimmune overlap syndromes [AIH and either PBC or PSC concurrently] were permitted entry); other CLDs that are not listed, or skin diseases associated with pruritus, including atopic dermatitis, chronic urticaria, eczema, lichen planus, lichen sclerosus, prurigo, or psoriasis (a detailed list of liver or skin diseases for exclusion were confirmed by a hepatologist); and/or had received a liver transplant

### Five-dimensional (5-D) itch scale

The 5-D itch scale is a self-administered, patient-reported outcome instrument designed to measure symptoms and impact of pruritus associated with many skin and tissue diseases.[1] The questionnaire has five domains that assess the following aspects of pruritus: duration (one item), degree (one item), direction (getting better or worse; one item), disability (extent of impact; four items), and distribution (locations on the body, one item). For distribution, sum of 0–2 body parts resulted in a score of 1, sum of 3–5 = score of 2, sum of 6–10 = score of 3, sum of 11–13 = score of 4, and sum of 14–16 = score of 5. All questions had a 2-week recall period. Response options were provided in the form of a 5-point Likert scale, ranging from less severe symptoms or impairments to more severe symptoms or impairments. The 5-D itch scale yields a global score and scores for each domain; higher scores indicate greater pruritus severity.

### 36-item short-form health survey (SF-36v2)

The SF-36v2 is a 36-item patient-reported outcome instrument that measures generic health-related quality of life in eight specific domains and two component summary indices: the physical and mental component summary.[2] The eight SF-36v2 domains include: physical functioning (10 items), role-physical (role limitations due to physical problems, four items), bodily pain (two items), general health perceptions (five items), vitality (four items), social functioning (two items), role-emotional (role limitations due to emotional problems, three items), and mental health (five items). This study used a 1-week recall period to align with the recall periods used by the other instruments in the survey. Item responses were aggregated into domain scores. Weighted summations of domain scores were used to calculate physical and mental component summary scores. The SF-36v2 domains and summary scores were all converted to a T-score metric (mean=50, standard deviation [SD]=10) that was standardised using a US general population normative sample derived from responses to a 2009 online normative survey conducted by QualityMetric.[2] Higher scores indicate better health outcomes for all domains and summary measures. To aid in interpretation, the norm-based scoring algorithm for the SF-36v2 utilised a linear T-score transformation where each domain and component summary measure had a mean of 50 and an SD of 10 in the general population.

### Full list of pruritus medications captured in the survey:

- Bile acid sequestrants / Anion-exchange resins (e.g., cholestyramine, colesevelam, colestipol, Cholestagel, Colestid, Questran, Welchol)
- Rifampicin/rifampin (e.g., Rifadin, Rimactane)
- Oral opiate antagonists (e.g., naltrexone, nalmefene, ReVia, Depade, Selincro)
- Selective serotonin reuptake inhibitors (SSRIs, e.g., sertraline, citalopram, fluoxetine, Zoloft, Celexa, Prozac)
- Gabapentin (e.g., Horizant, Gralise, Neuraptine, SmartRx Gaba-V Kit, Neurontin)
- Antihistamines (e.g., loratadine, fexofenadine, diphenhydramine, chlorpheniramine, brompheniramine, cetirizine, Alavert, Allegra, Benadryl, Claritin, Chlor-Trimeton, Dimetane, Piriton, Zyrtec)
- Phenobarbital (e.g., Solfoton)
- Topical remedies (e.g., creams, lotions, etc.)
- Plasmapheresis
- Albumin dialysis
- Molecular adsorbent recirculating system (MARS)
- Nasobiliary or external biliary drainage
- Light therapy

**Data protection**

All personal participant data collected and processed for the purposes of this research were managed with adequate precautions to ensure the confidentiality of those data, in accordance with applicable national and/or local laws and regulations on personal data protection. Access to data for GSK personnel was limited to authorised persons or roles, based on the principle of least privilege.

Confirmit, the web-based electronic data capture system used for the surveys, complied with GDPR requirements for data security, including 256-bit encryption, password protection, and role-based access. All data entries were dated and time stamped. Logic checks and verification processes (to identify inconsistent survey responses or unreasonably fast survey times) were employed to ensure data accuracy.

Data was transferred to QualityMetric (QM) via a secure transfer site. QM also employed functional and technical security processes to maintain the confidentiality of data. QM stored identifiable medical information in secure computer systems with strict access controls. Access to the data was permitted only as needed to perform company responsibilities. Data from the automated database could only be accessed through a secure protocol involving personal login information to establish the identity of the person accessing the data. Only staff approved for access were provided login access and data access activity was monitored.

**Original Survey Items**

The following questions are about **itch/pruritus**.

**Itch/pruritus**:

- Canfeel like a deep itch that can’t be relieved by scratching
- May go away for some time, but tends to come back
- May be linked to your liver disease
- Does **NOT** usually go away after scratching
- Is **NOT** caused by an insect bite or allergies (for example, to a plant like poison ivy or stinging nettles)

The next questions ask you to think back to when you **first experienced** **itch/pruritus**. For each question, please select the response that most closely corresponds to your own experience.

Compared to the time when your itch/pruritus began, how would you describe the severity of your itch/pruritus now?

 Much more severe now

 Somewhat more severe now

 About the same

 Somewhat less severe now

 Much less severe now

Compared to the time when your itch/pruritus began, would you say your itch/pruritus happens more or less frequently now?

 Much more frequently now

 Somewhat more frequently now

 About the same

 Somewhat less frequently now

 Much less frequently now

The next few questions ask you to think about your experience **in the time since your itch/pruritus began**.For each question, please select the response that most closely corresponds to your own experience.

In the time since your itch/pruritus began, please select any adjustments to your lifestyle that you had to make due to your itch/pruritus. Select all that apply.

 Moved to find a more favorable climate

 Changed vacation plans to find more favorable weather

 Changed choices in fashion or clothing

 Changed home climate control systems (heating, air conditioning, humidifier)

 Changed jobs or required some sort of adjustment/change in the workplace

 Stopped working altogether

 Changed diet

 Changed alcohol drinking habits

 Were unable to do daily chores/tasks

 Spent less time on hobbies or leisure time activities

 Other ________________________

 None of the above

In the time since your itch/pruritus began, please indicate how much each of the following makes your itch/pruritus worse.

|  | Not applicable | Not at all | Slightly worse | Somewhat worse | Much worse | Very much worse |
| --- | --- | --- | --- | --- | --- | --- |
| Heat / warm weather |  | 1  | 2  | 3  | 4  | 5  |
| Cold / cold weather |  | 1  | 2  | 3  | 4  | 5  |
| Certain types of clothes |  | 1  | 2  | 3  | 4  | 5  |
| Dry skin |  | 1  | 2  | 3  | 4  | 5  |
| Certain foods |  | 1  | 2  | 3  | 4  | 5  |
| Stress |  | 1  | 2  | 3  | 4  | 5  |
| Alcohol |  | 1  | 2  | 3  | 4  | 5  |
| Menstrual cycle |  | 1  | 2  | 3  | 4  | 5  |
| Other _____________ |  | 1  | 2  | 3  | 4  | 5  |

At any time since your itch/pruritus began, have you used any of the following for your itch/pruritus? Select all that apply.

 Bile acid sequestrants / Anion-exchange resins

(e.g., cholestyramine, colesevelam, colestipol, Cholestagel, Colestid, Questran, Welchol)

 Rifampicin / rifampin (e.g., Rifadin, Rimactane)

 Oral opiate antagonists

(e.g., naltrexone, nalmefene, ReVia, Depade, Selincro)

** **Selective serotonin reuptake inhibitors(SSRIs)

(e.g., sertraline, citalopram, fluoxetine, Zoloft, Celexa, Prozac)

 Gabapentin (e.g., Horizant, Gralise, Neuraptine, SmartRx Gaba-V Kit, Neurontin)

 Antihistamines

(e.g., loratadine, fexofenadine, diphenhydramine, chlorpheniramine, brompheniramine, cetirizine, Alavert, Allegra, Benadryl, Claritin, Chlor-Trimeton, Dimetane, Piriton, Zyrtec)

 Phenobarbital (e.g., Solfoton)

 Topical remedies (e.g., creams, lotions, etc.)

 Plasmapheresis

 Albumin dialysis

 Molecular adsorbent recirculating system (MARS)

 Nasobiliary or external biliary drainage

 Light therapy

 No treatment

 Other

Are you currently using any of the following for your itch/pruritus? Select all that apply.

*(offer only items endorsed in previous item)*

 Bile acid sequestrants / Anion-exchange resins

(e.g., cholestyramine, colesevelam, colestipol, Cholestagel, Colestid, Questran, Welchol)

 Rifampicin / rifampin (e.g., Rifadin, Rimactane)

 Oral opiate antagonists

(e.g., naltrexone, nalmefene, ReVia, Depade, Selincro)

** **Selective serotonin reuptake inhibitors(SSRIs)

(e.g., sertraline, citalopram, fluoxetine, Zoloft, Celexa, Prozac)

 Gabapentin (e.g., Horizant, Gralise, Neuraptine, SmartRx Gaba-V Kit, Neurontin)

 Antihistamines

(e.g., loratadine, fexofenadine, diphenhydramine, chlorpheniramine, brompheniramine, cetirizine, Alavert, Allegra, Benadryl, Claritin, Chlor-Trimeton, Dimetane, Piriton, Zyrtec)

 Phenobarbital (e.g., Solfoton)

 Topical remedies (e.g., creams, lotions, etc.)

 Plasmapheresis

 Albumin dialysis

 Molecular adsorbent recirculating system (MARS)

 Nasobiliary or external biliary drainage

 Light therapy

 No treatment

 Other

In the time since your itch/pruritus began, please indicate how much each of the following improves your itch/pruritus.

|  | Have not tried | Not at all | Slightly improved | Somewhat improved | Much improved | Very much improved |
| --- | --- | --- | --- | --- | --- | --- |
| Something cool on the skin |  | 1  | 2  | 3  | 4  | 5  |
| Over-the-counter topical creams, moisturizers, etc. |  | 1  | 2  | 3  | 4  | 5  |
| Prescription medications |  | 1  | 2  | 3  | 4  | 5  |
| Over-the-counter antihistamines |  | 1  | 2  | 3  | 4  | 5  |
| Scratching |  | 1  | 2  | 3  | 4  | 5  |
| Other _____________ |  | 1  | 2  | 3  | 4  | 5  |

In the time since your itch/pruritus began, how would you describe the sensation of your itch/pruritus? Select all that apply.

 Bugs crawling

 Relentless

 Want to tear my skin off

 Prickly/ needles

 Deep itch

 Burning

 Itch until I bleed

 Hives

 Urgent itch

 Itch feels like it is “in the veins”

 Scratching does not help

 Other ______________

In the time since your itch/pruritus began, in general, at what time of day has your itch/pruritus been the worst?

 During the day

 During the night

 About the same during the day and night

In the time since your itch/pruritus began, during which season is your itch/pruritus worst? Select all that apply.

 Spring

 Summer

 Fall/Autumn

 Winter

 My itch/pruritus is the same across seasons

 Don’t know

In the time since your itch/pruritus began, how has your healthcare provider approached your itch/pruritus? Select all that apply.

 Has not talked to me about my itch/pruritus

 Talked to me about my itch/pruritus

 Gave me questionnaires about my itch/pruritus

 Looked at my skin

 Performed biopsy of my skin / took skin sample

 Prescribed medication for my itch/pruritus

 Other ______________

The next questions ask you to think about your experience with **itch/pruritus** over the **past 2 weeks.** For each question, please select the response that most closely corresponds to your own experience.

Over the past 2 weeks, on average, how often did you experience itch/pruritus?

 Not at all

 One day per week

 Two or three days per week

 More than half the days (4-6 days per week)

 Every day

Over the past 2 weeks, how often have you scratched so much that it made your skin raw?

 Not at all

 One day per week

 Two or three days per week

 More than half the days (4-6 days per week)

 Every day

Over the past 2 weeks, when your itch/pruritus was at its worst, please indicate how much each aspect of your life was impacted by itch/pruritus:

|  | No impact | Very little impact | Some impact | Quite a bit of impact | A great deal of impact |
| --- | --- | --- | --- | --- | --- |
| Intimate relationships | 1  | 2  | 3  | 4  | 5  |
| Emotional well-being | 1  | 2  | 3  | 4  | 5  |
| Ability to concentrate | 1  | 2  | 3  | 4  | 5  |

Are you currently on a liver transplant list?

 Yes

 No

Has your healthcare provider indicated that you have cirrhosis?

 Yes

 No

Have you ever been diagnosed with COVID-19?

 Yes

 No

*(If “yes”, go on to subitem)*

When were you diagnosed with COVID-19? MMM/YYYY

## References

1 Elman S, Hynan LS, Gabriel V, et al. The 5-D itch scale: a new measure of pruritus. *Br J Dermatol* 2010;162(3):587-93. doi: 10.1111/j.1365-2133.2009.09586.x. [published Online First: 2009/12/10]

2 Maruish ME. User's Manual for the SF-36v2 Health Survey: Quality Metric Incorporated 2011.
